# Faecal immunochemical tests (FIT) can help to rule out colorectal cancer in patients presenting in primary care with lower abdominal symptoms: a systematic review conducted to inform new NICE DG30 diagnostic guidance

**DOI:** 10.1186/s12916-017-0944-z

**Published:** 2017-10-24

**Authors:** Marie Westwood, Shona Lang, Nigel Armstrong, Sietze van Turenhout, Joaquín Cubiella, Lisa Stirk, Isaac Corro Ramos, Marianne Luyendijk, Remziye Zaim, Jos Kleijnen, Callum G. Fraser

**Affiliations:** 1 Kleijnen Systematic Reviews Ltd, Unit 6, Escrick Business Park, Riccall Road, Escrick, York, YO19 6FD UK; 2 University Medical Centre, De Boelelaan 1117, 1081HV Amsterdam, Netherlands; 3 Department of Gastroenterology, Complexo Hospitalario, Universitario de Ourense, Ourense, Spain; 4 Institute for Medical Technology Assessment, Erasmus University Rotterdam, Rotterdam, The Netherlands; 5 Institute of Health Policy and Management, Erasmus University Rotterdam, Rotterdam, The Netherlands; 6 School for Public Health and Primary Care (CAPHRI), Maastricht University, Maastricht, The Netherlands; 7 University of Dundee, Ninewells Hospital and Medical School, Dundee, DD1 9SY UK

## Abstract

**Background:**

This study has attempted to assess the effectiveness of quantitative faecal immunochemical tests (FIT) for triage of people presenting with lower abdominal symptoms, where a referral to secondary care for investigation of suspected colorectal cancer (CRC) is being considered, particularly when the 2-week criteria are not met.

**Methods:**

We conducted a systematic review following published guidelines for systematic reviews of diagnostic tests. Twenty-one resources were searched up until March 2016. Summary estimates were calculated using a bivariate model or a random-effects logistic regression model.

**Results:**

Nine studies are included in this review. One additional study, included in our systematic review, was provided as ‘academic in confidence’ and cannot be described herein.

When FIT was based on a single faecal sample and a cut-off of 10 μg Hb/g faeces, sensitivity estimates indicated that a negative result using either the OC-Sensor or HM-JACKarc may be adequate to rule out nearly all CRC; the summary estimate of sensitivity for the OC-Sensor was 92.1% (95% confidence interval, CI 86.9–95.3%), based on four studies (*n* = 4091 participants, 176 with CRC), and the only study of HM-JACKarc to assess the 10 μg Hb/g faeces cut-off (*n* = 507 participants, 11 with CRC) reported a sensitivity of 100% (95% CI 71.5–100%). The corresponding specificity estimates were 85.8% (95% CI 78.3–91.0%) and 76.6% (95% CI 72.6–80.3%), respectively.

When the diagnostic criterion was changed to include lower grades of neoplasia, i.e. the target condition included higher risk adenoma (HRA) as well as CRC, the rule-out performance of both FIT assays was reduced.

**Conclusions:**

There is evidence to suggest that triage using FIT at a cut-off around 10 μg Hb/g faeces has the potential to correctly rule out CRC and avoid colonoscopy in 75–80% of symptomatic patients.

**Systematic review registration:**

PROSPERO 42016037723

**Electronic supplementary material:**

The online version of this article (doi:10.1186/s12916-017-0944-z) contains supplementary material, which is available to authorized users.

## Background

Colorectal cancer (CRC) is the third most common cancer in the UK population overall and in people aged 50 years and over, after breast and lung cancer for females and prostate and lung cancer for males. The Office for National Statistics (ONS) cancer registration data for 2013 showed approximately 35,000 new cases of CRC in England (18,839 males and 14,926 females) [1]. CRC accounted for approximately 11.5% of all new cancers diagnosed in 2013 (12.6% in males and 10.4% in females) and increased with age to 14.2% of cancers in males and 15.2% in females aged 80 years and over [1].

The UK has established bowel screening programmes with colonoscopy being offered following a positive faecal occult blood test using a guaiac test or qualitative faecal immunochemical test (FIT). Screening is offered to people between the ages of 60 and 74 years in England, Wales and Northern Ireland, and between the ages of 50 and 74 years in Scotland. Older people can opt to continue screening. Despite efforts to promote screening, the 2015 National Bowel Cancer Audit Report stated that, of all patients diagnosed with CRC in 2014, 55% were diagnosed following a referral by a general practitioner (GP), 9% (20% of those in the eligible age range for screening) were diagnosed through the National Health Service (NHS) Bowel Cancer Screening Programme and 20% were only diagnosed following an emergency presentation (referral source data were missing for 16% of patients) [2]. The Cancer Audit Report recommended work to promote awareness of CRC symptoms, as well as work to promote screening uptake; however, increased awareness of symptoms and consequent presentation in primary care could result in more invasive investigations such as colonoscopy being required. Estimates from the charity Bowel Cancer UK [3] have suggested that there will be a 10–15% year-on-year increase in demand for colonoscopies, which would have an impact on the 2-week suspected cancer referral time applied in England and NHS capacity [4]. Colonoscopy has associated risks which include bowel perforation, bleeding and abdominal pain [5]; UK NHS audit data have provided an estimated rate of complications (perforations and significant haemorrhages) of approximately 3 per 1000 colonoscopies [6]. A recent review reported that most colonoscopies performed in symptomatic patients do not find either CRC or other serious bowel disease and do not result in changes to the treatment approach [7]. The identification of tests which can help to select those people with symptoms who are more likely to benefit from further investigation is an important goal for optimal use of colonoscopy.

In addition to the 2-week wait referral criteria, the 2015 version of the National Institute for Health and Care Excellence (NICE) guideline ‘Suspected cancer: recognition and referral’ (NG12) recommended tests for occult blood in faeces in adults without rectal bleeding who are aged 50 years and over and have unexplained abdominal pain or weight loss; are aged under 60 years and have changes in their bowel habit or iron deficiency anaemia; or are aged 60 years and over and have anaemia in the absence of iron deficiency [4]. These recommendations were problematic in that they were widely interpreted as a recommendation for a traditional guaiac faecal occult blood test (gFOBT), a method which has relatively poor sensitivity (approximately 75%) [8–10] in symptomatic populations and which is no longer widely available in the UK NHS, outside the screening programmes. The recommendations were also criticised for not incorporating clinical judgement and hence potentially leading to high numbers of inappropriate referrals, particularly in younger people [11, 12].

It has been suggested that using quantitative FIT to select patients for referral has the potential to reduce unnecessary colonoscopies and provide more accurate classification of patients than traditional, symptoms-based guidelines [13]. FIT is recommended in European Commission screening guidelines [14] and has now been approved for use in the Scottish Bowel Screening Programme, the NHS Bowel Cancer Screening Programme in England and Bowel Screening Wales. It is vital to remember that evidence about the performance of FIT in asymptomatic population-based screening populations cannot be used to decide whether FIT should be recommended to inform referral decisions in people with symptoms suggestive of lower gastrointestinal tract disease, particularly CRC. This is because the prevalence of CRC may be higher in a population with low level symptoms than in the wider population who are eligible for screening. Furthermore, FIT used for screening applications may be qualitative analyses or use higher cut-off faecal haemoglobin concentrations than would be considered appropriate for the triage of people with symptoms.

This systematic review analysed the clinical effectiveness of FIT for triaging referrals in people with lower abdominal symptoms, particularly those who would be considered to be at low risk of having CRC. The review was undertaken as part of a diagnostic appraisal to inform the development of new NICE diagnostics guidance (DG30) [15]. The appraisal also included the development of a cost-effectiveness model, which is not included in this article [16].

## Methods

We conducted a systematic review with the main aim of summarising the evidence about the effectiveness of quantitative FIT for triage of people presenting with lower abdominal symptoms, where a referral to secondary care for investigation of suspected CRC is being considered, but the 2-week criteria are not met. Systematic review methods followed the principles outlined in the Handbook for Diagnostic Test Accuracy Reviews [17], the Centre for Reviews and Dissemination guidance for undertaking reviews in health care [18] and the NICE Diagnostic Assessment Programme manual [19].

### Data sources

The following databases were searched from inception to March 2016: MEDLINE; MEDLINE In-Process Citations and Daily Update; MEDLINE Epub Ahead of Print; Embase; Cochrane Database of Systematic Reviews (CDSR); Cochrane Central Register of Controlled Trials (CENTRAL); Database of Abstracts of Reviews of Effects (DARE); Health Technology Assessment (HTA) Database; NHS Economic Evaluation Database (NHS EED); International Network of Agencies for Health Technology Assessment (INAHTA); National Institute for Health Research (NIHR) Health Technology Assessment Programme; Aggressive Research Intelligence Facility (ARIF); PROSPERO. We also searched clinical trials registers (National Institutes of Health (NIH) ClinicalTrials.gov, European Union (EU) Clinical Trials Register and World Health Organization (WHO) International Clinical Trials Registry Platform) and conference proceedings (American Gastroenterological Association, Digestive Disease Week (DDW), Annual Meeting of the American Association for Clinical Chemistry and Laboratory Medicine (AACC), British Society of Gastroenterology (BSG) Annual Meeting, United European Gastroenterology Week (UEGW) and the European Congress of Clinical Chemistry and Laboratory Medicine (IFCC-EFLM), 2011–2015). Furthermore, we contacted experts in the field, with the aim of identifying any unpublished studies. Search strategies were based on index test (FIT assays) and target condition (CRC) and did not include any study design terms or filters [20]; example search strategies are provided online (Additional file 1: Material S1). No restrictions on language or publication status were applied to any searches.

### Inclusion criteria

Diagnostic cohort studies, which assessed the accuracy of quantitative FIT assays in people with lower abdominal symptoms who were being investigated for suspected CRC, were eligible for inclusion.

We included studies where the participant selection criteria were unclear, but where the population was described as symptomatic/suspected CRC and no asymptomatic participants were included. Where studies were conducted in mixed populations (both symptomatic and asymptomatic people included), study authors were contacted to request separate data for the symptomatic people sub-group. Studies conducted in people with pre-existing gastrointestinal tract co-morbidities were excluded.

Only clinical evaluations of the following quantitative FIT assays, which are commercially available in the UK, were included: OC-Sensor (Eiken Chemical Co. Ltd, Tokyo, Japan, supplied in the UK by MAST Group Ltd, Bootle, Merseyside); HM-JACKarc (Kyowa-Medex Co. Ltd, Tokyo, Japan, supplied in the UK by Alpha Laboratories Ltd, Eastleigh, Hants); FOB Gold (Sentinel Diagnostics, Milan, Italy, supplied in the UK by Sysmex UK Ltd, Milton Keynes); Ridascreen (R-Biopharm AG, Darmstadt, Germany, supplied in the UK by R-Biopharm Rhone Ltd, Glasgow).

Included studies were required to confirm diagnosis using colonoscopy as the reference standard and to report sufficient data to determine the numbers of true positive (TP), false positive (FP), false negative (FN) and true negative (TN) test results. Where studies reported FIT uptake rates or test accuracy data for other target conditions, in addition to CRC (e.g. adenoma, particularly higher risk, inflammatory bowel disease, organic bowel disease), we also included these data in our review.

Studies were screened for relevance independently by two reviewers, and full text articles of studies considered potentially relevant were assessed for inclusion by one reviewer and checked by a second. Disagreements, at either stage of study selection, were resolved through discussion and consensus, or by consultation with a third reviewer.

### Data extraction

One reviewer extracted data using a pre-study piloted data extraction form, and the extractions were checked by a second reviewer; any disagreements were resolved through discussion and consensus or by consultation with a third reviewer. Data were extracted on the following: study details, inclusion and exclusion criteria, participant characteristics (demographic characteristics, presenting symptoms, other CRC risk factors), target condition (CRC, advanced neoplasia (higher risk adenoma or CRC), other significant bowel disease outcomes (as reported)), details of the FIT test (manufacturer, analyser used, definition of cut-off faecal haemoglobin concentration (f-Hb), sampling procedure, detection method), details of the reference standard, definitions of the target conditions, test performance outcome measures (numbers of TP, FP, FN and TN test results) and proportion of study participants who returned a FIT sample (extracted as an indicator of acceptability).

### Quality assessment

The methodological quality of included studies was assessed using Quality Assessment of Diagnostic Accuracy Studies (QUADAS-2) [21], which uses four domains to assess risk of bias and three domains to assess the applicability of the study to the review question. Studies which reported the diagnostic performance of a risk prediction score that included FIT, in addition to measure of the accuracy of FIT alone, were additionally assessed using the prediction study risk of bias assessment tool (PROBAST) [22]. Quality assessment was undertaken by one reviewer and checked by a second reviewer, and any disagreements were resolved by consensus or discussion with a third reviewer.

### Analysis

Sensitivity and specificity, with 95% confidence interval (CI), were calculated for each set of 2 × 2 data. The bivariate/hierarchical summary receiver operating characteristic (HSROC) model was used to estimate summary sensitivity and specificity with 95% CI and prediction regions around the summary points and to derive HSROC curves for meta-analyses involving four or more studies [23–25]. This approach allows for between-study heterogeneity in sensitivity and specificity and for the trade-off (negative correlation) between sensitivity and specificity commonly seen in diagnostic meta-analyses. For meta-analyses with fewer than four studies, we estimated separate pooled estimates of sensitivity and specificity, using random-effects logistic regression [26]. Heterogeneity was assessed visually using summary ROC plots and statistically using the variance of logit (sensitivity) and logit (specificity), where “logit” indicates the logistic function: the smaller these values, the less heterogeneity between studies. Analyses were performed in Stata 10 (StataCorp LP, College Station, TX, USA), using the *metandi* command. For analyses that would not run in Stata, we used Meta-DiSc [27].

Studies were grouped by FIT assay type, by target condition and by cut-off f-Hb. Stratified results tables and ROC space plots are presented to illustrate the variation of test performance by cut-off f-Hb, and flow charts are provided to illustrate the progress of a hypothetical cohort of patients through a diagnostic work-up that includes triage using FIT at the optimal cut-off.

## Results

### Overview of included studies

**Fig. 1:**
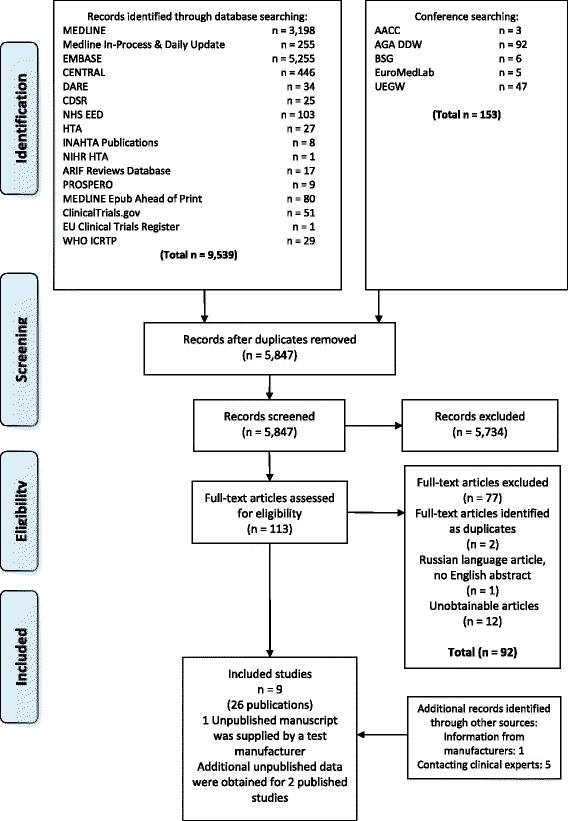
Flow of studies through the review process

The searches identified 5782 references; nine studies, reported in 26 publications [28–53], were included in our review. A table detailing the primary and related publications, for each included study, is provided online (Additional file 2: Table S1). One additional unpublished study was provided by Sysmex UK Ltd (the supplier of FOB Gold reagents for FIT assays in the UK, manufactured by Sentinel Diagnostics, Milan, Italy). This study was included in the version of our full report, which was considered by the NICE Diagnostics Appraisal Committee when formulating guidance, but it cannot be included in this article because it was provided as ‘academic in confidence’. Additional unpublished data were supplied by the authors of two studies [33, 35]. Figure 1 shows the flow of studies through the review process. Full details of the studies excluded after full text analysis, with reasons for exclusion, are provided online (Additional file 3: Table S2).

Five studies reported accuracy data for the OC-Sensor FIT assay (Eiken Chemical Co. Ltd, Tokyo, Japan); one used the io analyser [28], one used the Diana automated immunoturbidimetric analyser [32], two used the MICRO desktop analyser [29, 35] and one did not report the analyser used [31]. Three studies reported accuracy data for the HM-JACKarc automated system (Kyowa Medex Co. Ltd, Tokyo, Japan) [33, 34, 52]. The remaining two studies reported accuracy data for the FOB Gold assay; one used the Roche Modular P/917 analyser (Roche Diagnostics Ltd, West Sussex, UK) [30], and the un-published study, provided as ‘academic in confidence,’ and not included here, used the SENTiFIT 270 analyser (Sentinel Diagnostics, Milan, Italy). Five studies reported receiving some funding from manufacturers (including supply of test kits, reagents and analysers) [31–34, 52], one study did not report details of funding [30] and the unpublished study was conducted at the request of the test manufacturer. No studies were identified which assessed the diagnostic performance of RIDASCREEN Hb or RIDASCREEN Hb/Hp complex in symptomatic patients.

### Study quality

All studies included in this systematic review were diagnostic cohort studies (i.e. studies conducted in a group of patients in whom the presence of the target condition is suspected and which are therefore representative of the setting in which the test would be used in practice); diagnostic case-control studies (i.e. studies in which a test is evaluated in healthy controls and people with a previously established diagnosis of the target condition) were excluded. The methodological quality of these studies was assessed using the QUADAS-2 tool [21]. Two studies were reported only as conference abstracts, with limited descriptions of methods [30, 52], and two studies were rated as having ‘low’ risk of bias for all domains [31, 34]. Three studies were rated as ‘high’ risk of bias on the flow and timing domain [28, 32, 52], because some patients who returned a sample for FIT (11–38%) were subsequently excluded from the analyses. All of the included studies were rated as having ‘high’ concerns about applicability to the specific research aim with respect to participants. This happened because all studies included some participants who had symptoms that may be considered to be associated with a higher probability of CRC and which are components of the criteria for 2-week referral as defined in NG12 [4] (e.g. rectal bleeding). In addition, only one study was conducted in a primary care setting, reporting that FIT was requested by GPs at the point of referral to secondary care [28]. The results of the QUADAS-2 assessment are summarised in Table 1, and full details of the participant characteristics, FIT assay and reference standard for each study are provided online (Additional file 4: Table S3). PROBAST assessments for the two studies that reported the development and validation of risk prediction scores [29, 50] are provided online (web-Additional file 5: Table S4).

**Table 1:** QUADAS-2 results for studies of FIT assays

Study	Risk of bias				Applicability concerns		
	Patient selection	Index test	Reference standard	Flow and timing	Patient	Index test	Reference standard
Auge 2016 [34]	Low	Low	Low	Low	High	Low	Low
Cubiella 2014 [31]	Low	Low	Low	Low	High	Low	Low
Godber 2016 [33]	Low	Low	Unclear	Low	High	Low	Low
Krivec 2011 [30]	Unclear	High	Unclear	Unclear	High	Low	High
McDonald 2013 [32]	Low	Low	Unclear	High	High	Low	Unclear
Mowat 2015 [28]	Low	Low	Low	High	High	Low	Low
Rodríguez-Alonso 2015 [29]	Unclear	Low	Low	Low	High	Low	Low
Terhaar sive Droste 2011 [35]	Low	Low	Low	Unclear	High	Low	Low
Thomas 2016 [52]	Unclear	Low	Unclear	High	High	Low	Low

### Diagnostic performance of the OC-Sensor FIT assay

**Fig. 2:**
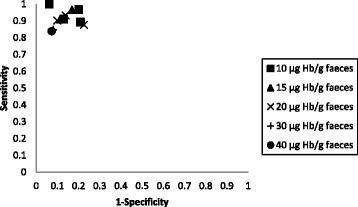
ROC space plot for the OC-Sensor assay using different faecal haemoglobin cut-offs for the target condition of CRC

All five studies that evaluated the OC-Sensor assay reported accuracy data, where CRC was the specified target condition [28, 29, 31, 32, 35]. The prevalence of CRC, diagnosed at colonoscopy, in these studies ranged from 2.1 to 12.3%. Four studies [28, 29, 31, 32] also reported data for the composite target condition of advanced neoplasia (AN) defined as CRC or higher risk adenoma (HRA), or CRC or advanced adenoma; where a definition was provided, an HRA was defined as an adenoma ≥ 10 mm in diameter or three or more adenomas of any size: advanced adenomas were considered as adenomas > 10 mm in diameter or adenomas with villous architecture or high grade dysplasia [29, 31]. Three studies reported additional accuracy data on various non-malignant and composite target conditions [28, 32, 35]. Accuracy data, for all target conditions and cut-offs evaluated, are summarised in Table 2, and accuracy data for CRC at all f-Hb cut-offs evaluated are summarised in Fig. 2.

**Table 2:** Accuracy of the OC-Sensor FIT assay

Study	Prevalence of condition specified below (%)	f-Hb cut-off (μg Hb/g faeces)	True positive	False negative	False positive	True negative	Total	Negative predictive value % (95% CI)	Sensitivity % (95% CI)	Specificity % (95% CI)
Target condition CRC										
10 μg Hb/g faeces or equivalent										
McDonald 2013 [32]	2.1	≥10^a^	6	0	17	257	280	100 (98.5, 100)	100 (54.1, 100)	93.8 (90.3, 96.3)
Mowat 2015 [28]	3.7	≥10	25	3	151	571	750	99.5 (98.5, 99.8)	89.3 (71.8, 97.7)	79.1 (75.9, 82.0)
Rodríguez-Alonso 2015 [29]	3.0	≥10	29	1	196	777	1003	99.9 (99.3, 100)	96.7 (82.8, 99.9)	79.9 (77.2, 82.3)
Terhaar sive Droste 2011 [35]	5.4	≥10^a^	102	10	253	1693	2058	99.4 (98.9, 99.7)	91.1 (84.2, 95.6)	87.0 (85.4, 88.5)
Summary estimate									**92.1 (86.9, 95.3)**	**85.8 (78.3, 91.0)**
15 μg Hb/g faeces or equivalent										
Rodríguez-Alonso 2015 [29]	3.0	≥15	29	1	164	809	1003	99.9 (99.3, 100)	96.7 (82.8, 99.9)	83.1 (80.6, 85.4)
Terhaar sive Droste 2011 [35]	5.4	≥15^a^	102	10	219	1727	2058	99.4 (98.9, 99.7)	91.1 (84.2, 95.6)	88.7 (87.3, 90.1)
Summary estimate									**92.3 (86.6, 96.1)**	**86.9 (85.6, 88.1)**
20 μg Hb/g faeces or equivalent										
Cubiella 2014 [31]	12.3	≥20^a^	85	12	156	534	787	97.8 (96.2, 98.7)	87.6 (79.0, 93.2)	77.4 (74.0, 80.4)
Rodríguez-Alonso 2015 [29]	3.0	≥20	28	2	135	838	1003	99.8 (99.1, 99.9)	93.3 (77.9, 99.2)	86.1 (83.8, 88.2)
Terhaar sive Droste 2011 [35]	5.4	≥20^a^	101	11	193	1753	2058	99.4 (98.9, 99.7)	90.2 (83.1, 95.0)	90.1 (88.7, 91.4)
Summary estimate									**89.5 (84.9, 93.1)**	**86.6 (85.4, 87.7)**
Other f-Hb cut-offs										
Terhaar sive Droste 2011 [35]	5.4	≥30^a^	95	17	158	1788	2058	99.1 (98.5, 99.4)	84.8 (76.8, 90.9)	91.9 (90.6, 93.1)
Terhaar sive Droste 2011 [35]	5.4	≥40^a^	94	18	142	1804	2058	99.0 (98.4, 99.4)	83.9 (75.8, 90.2)	92.7 (91.5, 93.8)
Target condition advanced neoplasia (CRC or HRA)										
10 μg Hb/g faeces or equivalent										
McDonald 2013 [32]	10.4	≥10^a^	17	12	6	245	280	95.3 (92.0, 97.3)	58.6 (38.9, 76.5)	97.6 (94.9, 99.1)
Mowat 2015 [28]	9.1	≥10	45	23	131	551	750	96.0 (94.1, 97.3)	66.2 (53.7, 77.2)	80.8 (77.6, 83.7)
Rodríguez-Alonso 2015 [29]	13.3	≥10	82	51	144	726	1003	93.4 (91.5, 95.0)	61.7 (52.8, 69.9)	83.4 (80.8, 85.9)
Summary estimate									**62.6 (56.0, 68.9)**	**84.4 (82.7, 86.1)**
20 μg Hb/g faeces or equivalent										
Cubiella 2014 [31]	22.5	≥20^a^	127	50	114	496	787	90.8 (88.1, 93.0)	71.8 (64.4, 78.1)	81.3 (77.9, 84.3)
Rodríguez-Alonso 2015 [29]	13.3	≥20	71	62	92	778	1003	92.6 (90.7, 94.2)	53.4 (44.5, 62.1)	89.4 (87.2, 91.4)
Summary estimate									**63.9 (58.2,69.2)**	**86.1(84.2, 87.8)**
Other f-Hb cut-offs										
Rodríguez-Alonso 2015 [29]	13.3	≥15	76	57	117	753	1003	93.0 (91.0, 94.5)	57.1 (48.3, 65.7)	86.6 (84.1, 88.7)
Target condition all neoplasia (CRC, HRA or low risk adenoma)										
McDonald 2013 [32]	21.4	≥10^a^	35	25	3	217	280	89.7 (85.2, 92.9)	58.3 (44.9, 70.9)	98.6 (96.1, 99.7)
Target condition significant bowel disease (CRC, HRA or IBD)										
Mowat 2015 [28]	13.6	≥10	70	32	106	542	750	94.4 (92.2, 96.0)	68.6 (58.7, 77.5)	83.6 (80.6, 86.4)
Target condition significant bowel disease (CRC, HRA, low risk adenoma or IBD)										
McDonald 2013 [32]	30.7	≥10^a^	49	37	2	192	280	83.8 (78.5, 88.0)	57.0 (45.8, 67.6)	99.0 (96.3, 99.9)

^a^Converted from ng Hb/ml buffer using a multiplication factor of 0.2 [67, 68]

*CRC* colorectal cancer, *f-Hb* faecal haemoglobin, *HRA* higher risk adenoma, *IBD* inflammatory bowel disease

**Fig. 3:**
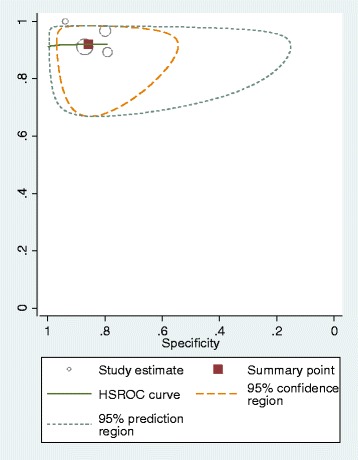
HSROC for the OC-Sensor assay using a 10 μg Hb/g faeces cut-off and a single sample (four studies)

The optimal test performance (maximising both sensitivity and specificity) appeared to occur with f-Hb cut-offs of 10 or 15 μg Hb/g faeces, with most data being available for the 10 μg Hb/g faeces cut-off. The summary estimates of sensitivity and specificity, using the 10 μg Hb/g faeces cut-off, were 92.1% (95% CI 86.9–95.3%) and 85.8% (95% CI 78.3–91.0%), respectively, based on data from four studies [28, 29, 32, 35]. Figure 3 shows the HSROC for the OC-Sensor assay, using the 10 μg Hb/g faeces cut-off, based on these four studies. As can be seen from Fig. 3 and Table 2, between-study heterogeneity was greater for specificity values than for sensitivity values; the coefficient of variance of logit sensitivity was 0.0002362 (standard error 0.0145951) and the coefficient of variance of logit specificity was 0.2577195 (standard error 0.2096304).

**Fig. 4:**
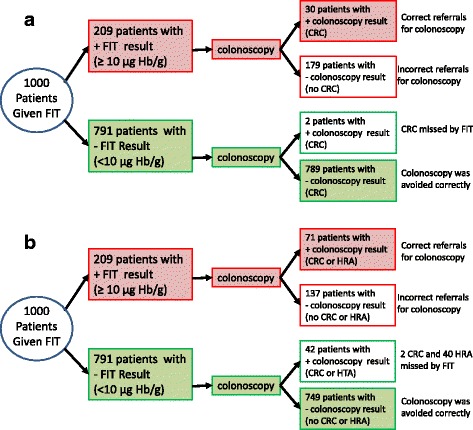
Testing outcomes for a hypothetical cohort of 1000 patients using OC-Sensor at the 10 μg Hb/g faeces threshold, for the target condition **a** CRC and **b** AN

Three studies reported separate accuracy data, using the 10 μg Hb/g faeces cut-off, for both CRC and the composite target condition AN [28, 29, 32]. The prevalence of CRC across these studies was 3.1%. Using test performance data from these three studies, and a CRC prevalence estimate of 3.1% to consider the outcome of testing for a hypothetical cohort of 1000 patients, the results indicate that, using the 10 μg Hb/g faeces cut-off, two CRCs would be missed and 179 unnecessary colonoscopies would be carried out (assuming that all patients with a positive FIT result receive colonoscopy and that all colonoscopies conducted in patients without CRC are considered unnecessary). CRC would be correctly ruled out by FIT, avoiding colonoscopy, in 789 of the 1000 patients (Fig. 4a). Expanding the target condition from CRC only to AN resulted in an increase in prevalence from 3.1 to 11.3% [28, 29, 32]. If the 10 μg Hb/g faeces cut-off were applied to the expanded target condition, for the hypothetical cohort of 1000 patients, the number of missed cases would increase from 2 to 42 (2 CRC and 40 HRA); using this cut-off, 137 unnecessary colonoscopies would be carried out and AN would be correctly ruled out in 749 of the 1000 patients (Fig. 4b). Approximately 22% of those classified as having a false positive FIT result for CRC would have HRA identified at colonoscopy. One study [28] evaluated the diagnostic performance of OC-Sensor (10 μg Hb/g faeces threshold) for a further composite target condition that included CRC and HRA plus inflammatory bowel disease (IBD). Results from this study (Table 2) indicate that 45 of the 151 participants (29.8%) who were classified as having false positive FIT results for CRC actually had other significant bowel pathology (HRA or IBD) and may thus have benefitted from secondary care investigation.

One of the three studies described above also conducted multivariable analysis, using forward conditional logistic regression modelling, with the aim of identifying independent predictors of CRC and AN [29]. The CRC analysis identified male gender (odds ratio, OR 2.39 (95% CI 1.039–5.519), *p* = 0.041), iron deficiency anaemia (OR 2.99 (95% CI 1.27–7.03), *p* = 0.012) and f-Hb ≥ 10 μg Hb/g faeces (OR 86.60 (95% CI 11.70–641.16), *p* < 0.001) as independent predictors [29]. The AN analysis identified male gender (OR 2.36 (95% CI 1.50–3.40), *p* < 0.001), age (OR 1.36 (95% CI 1.13–1.63), *p* < 0.001) and f-Hb ≥ 10 μg Hb/g faeces (OR 7.54 (95% CI 5.03–11.28), *p* < 0.001) as independent predictors; age was treated as a categorical variable in this model (≤40 years, 41–60 years, 51–60 years, 61–70 years, ≥ 70 years) [29]. The results of modelling were used to derive a risk score for AN; the scoring system assigned integer values to each independent predictor based on their coefficients from the logistic regression model [29]. The score ranged from 0 to 11 with points assigned as follows: age < 40 years = 0 points, age 41 to 50 years = 1, age 51 to 60 years = 2, age 61 to 70 years = 3, age > 70 years = 4; female gender = 0, male gender = 2; f-Hb < 10 μg Hb/g faeces = 0, f-Hb ≥ 10 μg Hb/g faeces = 5 [29]. The model was validated using a split sampling technique (data from 680 study participants (67.8%) were used to develop the model and data from 323 participants (32.2%) were used for validation) [29]. In the validation sample, a risk score ≥ 5 had a sensitivity for AN of 88.1% (95% CI 74.3–96.0%) and a specificity of 63.3% (95% CI 57.4–69.0%) [29]. We identified a second risk score for CRC in symptomatic patients, based on FIT age and sex (the Faecal haemoglobin, Age and Sex Test (FAST) score) [53], which was developed as a simplification of the COLONPREDICT model approach [50]. The logistic regression model used to develop the FAST score included gender, age as a continuous variable and f-Hb as a categorical variable (0, 0 to < 20, 20 to 200, and ≥ 200 μg Hb/g faeces [53]. The validation cohort for this model used data from five studies included in this systematic review [28, 29, 32, 33, 50], incorporating data from a number of FIT assays, including OC-Sensor and HM-JACKarc [28, 29, 32, 33], and an additional cohort recruited to the COLONPREDICT study between March 2014 and March 2015 [50]. The example FAST score cut-offs used to assess the performance in the validation cohort corresponded to the beta coefficients of the FAST score with 90% and 99% sensitivity in the development cohort (4.50 and 2.12, respectively. In the validation cohort, a FAST score of ≥ 4.50 had a sensitivity of 89.3% (95% CI 84.1–93.0%) and a specificity of 82.3% (95% CI 81.1–83.5%) for CRC. In order to avoid missing any CRC, a lower FAST score cut-off of ≥ 2.12 was required; the sensitivity and specificity estimates at this cut-off were 100% (95% CI 97.7–100%) and 19.8% (95% CI 18.6–21.1%), respectively, for CRC and 96.7% (95% CI 94.9–98.0%) and 21.5% (95% CI 20.1–22.9%) for AN [50].

Four studies reported information about uptake rates in participants invited to provide a sample for FIT [28, 29, 31, 32]. The proportion of people invited to participate in FIT who return a faecal sample can be regarded as a possible indicator of the acceptability of the test; however, the context in which patients were asked to provide a sample for FIT was also considered to be a key factor influencing uptake. Reported uptake rates for the OC-Sensor studies included in our review varied widely, ranging from 41% (in a study where patients were sent an invitation to participate along with their referral letter [32]) to 98% (in a study where patients were given the specimen collection device at their initial consultation with a gastroenterologist [29]): it is important to recognise that neither study was done in a primary care setting in which a GP would discuss the investigation with the patient and give a specimen collection device and associated literature at this time of consultation.

### Diagnostic performance of the HM-JACKarc FIT assay

Two of the three studies that evaluated the HM-JACKarc assay reported accuracy data, where CRC was the specified target condition [33, 52]. The prevalence of CRC diagnosed at colonoscopy in these studies was 2.2% [33] and 4.7% [52]. Only one study [33] also reported data for the composite target condition of AN (CRC or HRA). Two studies reported additional accuracy data for various non-malignant and composite target conditions [33, 52]. Accuracy data, for all target conditions and f-Hb cut-offs evaluated, are summarised in Table 3.

**Table 3:** Accuracy of the HM-JACKarc FIT assay

Study	f-Hb cut-off (μg Hb/g faeces)	Prevalence of condition specified below (%)	True positive	False negative	False positive	True negative	Total	Negative predictive value % (95% CI)	Sensitivity % (95% CI)	Specificity % (95% CI)
CRC										
Godber 2016 [33]	≥10	2.2	11	0	116	380	507	100 (99.0, 100)	100 (71.5, 100)	76.6 (72.6, 80.3)
Thomas 2016 [52]	≥7	5.1	21	2	89	338	450	99.4 (97.9, 99.8)	91.3 (72.0, 98.9)	79.2 (75.3, 83.0)
Target condition advanced neoplasia (CRC or HRA)										
10 μg Hb/g faeces										
Auge 2016 [34]	≥10	13.9	10	19	23	156	208	89.1 (83.7, 92.9)	34.5 (19.9, 52.7)	87.2 (81.6, 91.3)
Godber 2016 [33]	≥10	5.9	21	9	106	371	507	97.6 (95.6, 98.7)	70.0 (50.6, 85.3)	77.8 (73.8, 81.4)
Other f-Hb cut-offs										
Auge 2016 [34]	≥20	13.9	9	20	13	166	208	89.2 (84.0, 92.9)	31.0 (17.3, 49.2)	92.8 (88.0, 95.7)
Auge 2016 [34]	≥30	13.9	9	20	12	167	208	89.3 (84.1, 93.0)	31.0 (17.3, 49.2)	93.3 (88.7, 96.1)
Auge 2016 [34]	≥40	13.9	8	21	11	168	208	88.9 (83.6, 92.6)	27.6 (14.7, 45.7)	93.9 (89.4, 96.6)
Target condition significant bowel disease (CRC, HRA or IBD)										
Thomas 2016 [52]	≥7	NC	NC	NC	NC	NC	450	96.5 (94.5, 98.4)	72.1 (58.7, 85.5)	80.6 (76.7, 84.4)
Target condition significant bowel disease (CRC, HRA, IBD or colitis)										
Godber 2016 [33]	≥10	9.3	32	13	90	349	484	96.4 (94.0, 97.9)	68.9 (53.2, 81.4)	80.2 (76.1, 83.7)
Godber 2016 [33]	≥15	9.3	31	14	77	362	484	84.6 (75.8, 90.6)	66.7 (50.9, 79.6)	83.1 (79.2, 86.5)
Godber 2016 [33]	≥20	9.3	29	16	63	376	484	79.7 (69.6, 87.1)	64.4 (48.7, 77.7)	85.7 (81.9, 88.7)
Godber 2016 [33]	≥25	9.3	29	16	55	384	484	77.5 (66.5, 85.6)	64.4 (48.7, 77.7)	87.5 (83.9, 90.3)
Godber 2016 [33]	≥30	9.3	29	16	50	389	484	75.8 (64.2, 84.5)	64.4 (48.7, 77.7)	88.6 (85.2, 91.4)
Godber 2016 [33]	≥35	9.3	29	16	47	392	484	74.6 (62.7, 83.7)	64.4 (48.7, 77.7)	89.2 (85.9, 92.0)
Godber 2016 [33]	≥40	9.3	29	16	44	395	484	73.3 (61.0, 82.9)	64.4 (48.7, 77.7)	90.0 (86.7, 92.5)

*CRC* colorectal cancer, *f-Hb* faecal haemoglobin, *HRA* higher risk adenoma, *IBD* inflammatory bowel disease, *NC* not calculable

**Fig. 5:**
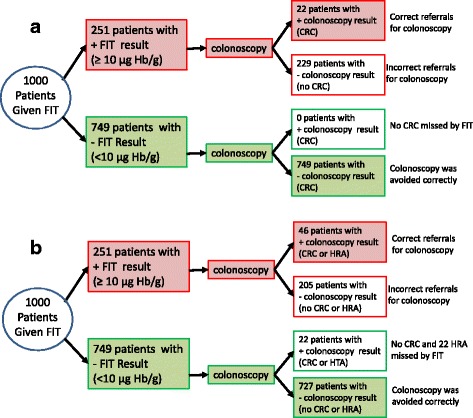
Testing outcomes for a hypothetical cohort of 1000 patients using HM-JACKarc at the 10 μg Hb/g faeces threshold, for the target condition **a** CRC and **b** AN

The optimal test performance (maximising both sensitivity and specificity) appeared to occur at the f-Hb cut-off of 10 μg Hb/g faeces. The estimates of sensitivity and specificity at this cut-off, derived from a single study, were 100% (95% CI 71.5–100%) and 76.6% (95% CI 72.6–80.3%), respectively [33]. Using accuracy and prevalence data from this study and the 10 μg Hb/g faeces cut-off to consider the outcome of testing for a hypothetical cohort of 1000 patients indicates that no CRC would be missed, but 229 unnecessary colonoscopies would be carried out (assuming that all patients with a positive FIT result receive a colonoscopy and that all colonoscopies conducted in patients without CRC are considered unnecessary); CRC would be correctly ruled out by FIT, avoiding colonoscopy, in 749 of the 1000 patients (Fig. 5a). Expanding the target condition from CRC only, to include CRC or HRA, resulted in an increase in prevalence from 2.2 to 5.9% [33]. If the 10 μg Hb/g faeces cut-off were applied to the expanded target condition, for the hypothetical cohort of 1000 patients, 22 cases of HRA would be missed, 205 unnecessary colonoscopies would be carried out and CRC and HRA would be correctly ruled out in 727 patients (Fig. 5b). Approximately 10% of those classified as having a false positive FIT result for CRC would have HRA identified at colonoscopy and a further 10% would be diagnosed with other significant bowel disease (IBD or colitis (Table 3)) [33]. Data from one study [34] indicated that the sensitivity of HM-JACKarc for AN was higher in men than in women and when the highest value from two consecutive faecal samples was used compared to using only the first sample; full results are provided online (Additional file 6: Table S5).

Two studies reported information about uptake rates in participants invited to provide a sample for FIT [33, 52]. The proportion of samples returned was higher (66%) in the study where information and collection devices were provided at an outpatient appointment [52] than in the study which sent collection devices and information by post (56%) [33].

### Diagnostic performance of the FOB Gold FIT assay

One study, reported in a conference abstract, assessed the performance of the FOB Gold FIT assay in symptomatic patients [30]. This study only reported data for the composite target condition of significant bowel disease, defined as cancer, polyps or bleeding; sensitivity and specificity were reported as 45.2% and 92.3%, using an f-Hb cut-off of 9 μg Hb/g faeces [30]. Insufficient information was provided to allow calculation of confidence intervals and 2 × 2 data. The unpublished study provided by Sysmex UK Ltd was considered by the NICE Diagnostics Appraisal Committee, when formulating the published recommendations, which include the FOB Gold assay [16].

## Discussion

### Statement of principal findings

All studies included in our systematic review were diagnostic cohort studies reporting accuracy data. When FIT was based on a single faecal sample and an f-Hb cut-off of 10 μg Hb/g faeces, sensitivity estimates indicated that a negative result using either the OC-Sensor or HM-JACKarc may be considered adequate to rule out most CRC. The summary estimate of sensitivity for the OC-Sensor was 92.1% (95% CI 86.9–95.3%), based on four studies [28, 29, 32, 35], and the negative predictive value varied between 99.4 and 100% across these studies. The only study of HM-JACKarc to assess the 10 μg Hb/g faeces cut-off reported a sensitivity of 100% (95% CI 71.5–100%). Where a lower diagnostic threshold was considered, i.e. the target condition included HRA as well as CRC, the rule-out performance of both FIT methods was reduced. Evidence suggests that risk scores may have the potential to provide a more reliable rule-out method than FIT alone at lower thresholds of disease, but that this is achieved at the cost of very poor specificity [50, 53]. Triage using FIT at an f-Hb cut-off of 10 μg Hb/g faeces has the potential to correctly rule out most CRC and avoid colonoscopy in 75–80% of symptomatic adults. In addition, the apparent relatively high number of FIT false positive results observed when the target condition is CRC may be mitigated by the detection of other bowel pathology in these patients. Because all of the included studies were conducted in patients for whom a referral to secondary care had already been made or was being considered, these estimates of the triage performance of FIT can be considered to have incorporated the judgement of primary care clinicians. The full potential benefits of FIT in symptomatic patients, including those relating to diagnoses other than CRC, remain unclear. This issue may be particularly important in younger patients, where the prevalence of CRC is lowest and other diagnoses, particularly IBD, are more likely.

### Strengths and weaknesses of study

Our study followed rigorous systematic review methodology, and our findings have informed the development of up-to-date guidance. The new NICE DG30 diagnostic guidance, now published, states: ‘The OC Sensor, HM-JACKarc and FOB Gold quantitative faecal immunochemical tests are recommended for adoption in primary care to guide referral for suspected colorectal cancer in people without rectal bleeding who have unexplained symptoms but do not meet the criteria for a suspected cancer pathway referral’ [15]. Limitations of our review include a lack of studies directly comparing the performance of different FIT assays; thus, all data included in our assessment describes the clinical effectiveness of individual FIT methods and not their comparative effectiveness. Three of the ten studies included in our systematic review were rated as ‘high’ risk of bias on the flow and timing domain, because some patients who returned a sample for FIT (11–38%) were subsequently excluded from the analyses. However, we note that the main issue with respect to study quality was the fact that no study reported data totally specific to the low risk, symptomatic population defined in the 2015 version of the NICE guideline for suspected cancer recognition and referral (NG12) [4]; all studies included some participants who had symptoms (e.g. rectal bleeding) that are considered to be associated with a higher probability of CRC and are components of the current criteria for 2-week wait suspected cancer referral. The prevalence of CRC is likely to differ between populations with different presenting symptoms, and it is well known that the prevalence of the target condition can affect estimates of test performance [54]. The median prevalence of CRC in those studies included in our review, which used the optimal f-Hb cut-off of 10 μg Hb/g faeces, was 3.7% (range 2.1–5.4%), compared to the estimate of 1.5% for the relevant symptomatic group used in NG12 [55]. There is insufficient information to determine whether this difference will affect the performance of FIT in primary care. However, it could be argued that the patients included in the studies in our review are representative of those for whom FIT would be useful in practice, irrespective of existing referral guidelines. A comparison of FIT to the NICE 2-week wait referral criteria was outside the scope of our study; however, two of the studies included in our systematic review did consider this issue [29, 50]. These studies reported the development and validation of risk prediction models for CRC [48] and AN [29]; f-Hb was identified as an independent predictor in both, and in both cases the final model demonstrated improved rule-out performance compared to the NICE 2-week wait referral criteria. The score based on the AN model had an optimum sensitivity of 88.1% (95% CI 74.3–96.0%) compared to 38.3% (95% CI 30.0–47.2%) for the NICE criteria [29]. This study also reported data indicating that FIT alone, at the f-Hb cut-off of 10 μg Hb/g faeces, could offer improved rule-out performance for CRC compared to the NICE 2-week wait referral criteria; the sensitivity estimate for FIT was 96.7% (95% CI 82.2– 99.9%), compared to 46.7% (28.3–65.7%) for the NICE criteria [29]. The FAST score had an optimal sensitivity of 99.5% (97.0–100%) compared to 68.2% (95% CI 61.5–74.3%) for the NICE referral criteria [50].

### Strengths and weaknesses in relation to other studies

Systematic reviews have previously been conducted which assessed the performance of various FIT assays in screening settings [56, 57]. However, given the potential for target condition prevalence to affect estimates of test performance [54], it is important to determine the diagnostic accuracy of FIT in the population of interest. We identified one large systematic review which assessed the value of symptoms and additional diagnostic tests for CRC, used in symptomatic patients in primary care [58]. However, the searches for this review were completed in 2008, and it included only three studies of quantitative FIT assays which examined asymptomatic people as well as symptomatic patients. Our systematic review is the first to assess the performance of quantitative FIT as a triage test in patients with symptoms and to consider the potential utility of applying FIT as part of a simple risk score.

### Unanswered questions and future research

Population data indicate that f-Hb varies with age and sex, being higher in men and the elderly [59, 60]. Further, a recent study conducted in Scotland found that f-Hb also increased with increasing levels of deprivation (measured using the Scottish Index of Multiple Deprivation), and that this trend remained after controlling for age and sex [61]. Thus, at any f-Hb cut-off, more men, more older people and more people in high deprivation groups are likely to have a positive result on FIT testing. The findings on deprivation have been confirmed in a recent study performed in England [62]. We did not identify any sub-group test performance data for the target condition CRC; however, one of the studies included in our systematic review compared the accuracy of a FIT assay (HM-JACKarc) in men and women [34] for the target condition AN. This study found that, at all f-Hb cut-offs, the observed sensitivity of HM-JACKarc was higher in men than in women and the observed specificity was similar for men and women [34]. This indicates that, at any given f-Hb cut-off, more women than men with CRC or HRA may be missed by using FIT as a triage test to determine referral to secondary care. Validation data for the FAST Score [50] indicated that there were no significant differences in the sensitivity of this tool between men and women, patients under 50 years of age and those who were 50 years or over and FIT analytical system used. More data are needed to adequately assess whether there are clinically relevant differences in test performance between men and women and between other clinically relevant sub-groups: such data are needed for all FIT assays.

The effects on FIT performance of using multiple faecal samples per patient remain unclear. One study [34] included in our systematic review compared single versus double sampling and asked patients to collect two consecutive faecal samples. This study reported that sensitivity for AN was increased (at all f-Hb cut-offs) when the highest value from two consecutive samples was used, compared to using only the first sample; FIT results were discordant in 39.2% of participants [34]. There is currently insufficient information about intra-individual variation in f-Hb over time to determine the clinical utility of multiple sampling.

The scope of our systematic review did not include evaluation of the performance characteristics of FIT when used in combination with other biomarkers. Two of the studies in our systematic review did compare FIT and calprotectin assays for detection of significant bowel disease and concluded that FIT alone had better rule-out performance [28, 52]. Combination testing, where a positive result was defined as both tests positive, provided increased specificity [52]. FIT, at an f-Hb cut-off of 7 μg Hb/g faeces, combined with faecal calprotectin, at a cut-off of 50 μg/g faeces, had sensitivity, specificity and negative predictive value (NPV) values of 69.6% (95% CI 50.8–88.4%), 92.5% (95% CI 90.0–95.0%) and 98.3% (97.0–99.5%), compared to 91.3% (79.8–100%), 79.2% (75.3–83.0%) and 99.4% (98.6–100%) for FIT alone [52]. However, where a positive result was defined as either test positive, the addition of faecal calprotectin to FIT offered no advantages [28]. The sensitivity, specificity and NPV values for any detectable f-Hb or faecal calprotectin ≥ 50 μg/g faeces were 100%, 23.3% and 100% compared to 100%, 43.4% and 100% for any detectable f-Hb. Following the evaluation reported here and the promulgation of the draft guidance from NICE [16, 63], a peer-reviewed publication on the performance of the HM-JACKarc [64] on 430 patients expanded on the preceding study [52]. The additional data confirmed that FIT at an f-Hb cut-off of 7 μg Hb/g faeces is sufficiently sensitive to exclude most CRC, with higher values in left-sided lesions. Faecal calprotectin in combination did not appear to provide additional diagnostic information [52]. In contrast, we identified one further study which did not meet the inclusion criteria for our systematic review because it used a FIT assay unavailable in the UK and Europe, but which reported data on the performance characteristics of FIT in combination with faecal calprotectin, M2-PK or both (where a positive result was defined as at least one test positive) for the target conditions CRC and HRA, as well as data on the performance characteristics of FIT alone [65]. Faecal calprotectin is an inflammatory marker, whilst M2-PK is a key enzyme in tumour metabolism [65]. This study found that, in all cases, the addition of at least one further test to FIT resulted in markedly increased sensitivity and decreased specificity. The sensitivity and specificity estimates for FIT alone and CRC were 61.7% (95% CI 47.4– 74.2%) and 88.8% (95% CI 84.1–92.3%); for the combination of FIT and faecal calprotectin these estimates were 90.9% (95% CI 78.8–96.4%) and 35.9% (95% CI 29.7–42.6%), for FIT and M2-PK, sensitivity and specificity were 91.5% (95% CI 80.1–96.6%) and 57.1% (95% CI 50.6–63.2%) and for all three markers they were 95.7% (85.7–98.8%) and 24.1% (18.8–30.2%) [65]. Although all sensitivity estimates were generally lower, this pattern was repeated where the target condition was AN [65]. A second study also found that combining faecal calprotectin with FIT (where a positive result was defined as either or both tests positive) resulted in increased sensitivity and decreased specificity for AN (92% (95% CI 82–97%) and 49% (95% CI 43–54%)) compared to FIT alone (74% (95% CI 62–83%) and 82% (95% CI 78–86%)) [66]. This study did not meet the inclusion criteria for this assessment because it used a qualitative FIT method. The effectiveness of combining other biomarkers with quantitative FIT (at the f-Hb cut-off at which FIT is likely to be used in practice) remains unclear.

## Conclusions

### Implications for clinicians and policy makers

There is evidence to suggest that triage using OC-Sensor or HM-JACKarc FIT, at an f-Hb cut-off of 10 μg Hb/g faeces, has the potential to correctly rule out CRC and avoid colonoscopy in 75–80% of symptomatic patients. The relatively high proportion of FIT false positive results observed when the target condition is CRC may be mitigated by the potential to detect other bowel pathology in these patients. However, the importance of clinical judgement cannot be overemphasised. All of the studies included in this review were conducted in symptomatic populations selected on the basis of a GP’s intention to refer rather than on the presence of a specific set of symptoms alone; overuse of FIT or blanket referral following a positive result has the potential to overwhelm colonoscopy services. There are currently no data on the comparative performance of different FIT assays in this population. Given the trade-off between ease of use/simplicity and diagnostic performance, the clinical value of using additional variables (e.g. symptoms and further diagnostic tests) to develop risk scores for CRC and/or other significant bowel disease is likely to require further investigation.

### What this paper adds

**Table:** 

***What is already known on this subject*** The NICE guideline on suspected cancer recognition and referral (NG12) recommends testing for occult blood in faeces for patients with specified symptoms associated with a ‘low risk’ of bowel cancer. This guideline has been widely interpreted as a recommendation for guaiac faecal occult blood tests and it does not include any statements about the importance of clinical judgement in deciding when to test in this population. Faecal immunochemical testing has been approved for use in the Scottish Bowel Screening Programme, the NHS Bowel Cancer Screening Programme in England and Bowel Screening Wales, and existing systematic review evidence supports this.
***What this study adds*** Triage using quantitative FIT, at a faecal haemoglobin concentration cut-off of 10 μg Hb/g faeces, has the potential to avoid colonoscopy in 75–80% of symptomatic patients for whom a general practitioner is considering a referral to secondary care, but who do not meet the criteria for 2-week wait suspected cancer referral. Secondary care referral following a positive FIT may facilitate the identification of other significant bowel pathology in patients who are found not to have lower gastrointestinal tract cancer.

## Additional files

Additional file 1: Material S1. Full search strategies for MEDLINE and Embase. (DOCX 17 kb)

Additional file 2: Table S1. Details of included studies and related publications. (DOCX 18 kb)

Additional file 3: Table S2. Details of excluded studies with rationale for exclusion. (DOCX 37 kb)

Additional file 4: Table S3. Data extraction tables. (DOCX 33 kb)

Additional file 5: Table S4. PROBAST results for studies reporting the development and validation of risk scores that included FIT (DOCX 16 kb)

Additional file 6: Table S5. Sub-group data for HM-JACKarc. (DOCX 19 kb)

## Abbreviations

CDSR: Cochrane Database of Systematic Reviews
CENTRAL: Cochrane Central Register of Controlled Trials
CI: Confidence interval
CRC: Colorectal cancer
EED: Economic Evaluation Database
FIT: Faecal immunochemical test(s)
FN: False negative
FOBT: Faecal occult blood test
FP: False positive
gFOBT: Guaiac faecal occult blood test
GP: General practitioner
Hb: Haemoglobin
Hp: Haptoglobin
HRA: High risk adenoma
HSROC: Hierarchical summary receiver operating characteristic
HTA: Health Technology Assessment
IBD: Inflammatory bowel disease
ICER: Incremental cost-effectiveness ratio
ILMA: Immunoluminometric assay
INAHTA: International Network of Agencies for Health Technology Assessment
NICE: National Institute for Health and Care Excellence
NIH: National Institutes of Health
NIHR: National Institute for Health Research
NPV: Negative predictive value
ONS: Office for National Statistics
ROC: Receiver operating characteristic
SROC: Summary receiver operating characteristic
TN: True negative
TP: True positive
