# Supplementary material for: Faecal immunochemical tests (FIT) can help to rule out colorectal cancer in patients presenting in primary care with lower abdominal symptoms: a systematic review conducted to inform new NICE DG30 diagnostic guidance
Source: BMC Med. 2017 Oct 24;15:189. doi: 10.1186/s12916-017-0944-z (PMC5654140; doi:10.1186/s12916-017-0944-z)
Supplement: Supplementary file 1 — Full search strategies for MEDLINE and Embase. (DOCX 17 kb) [file 12916_2017_944_MOESM1_ESM.docx]

**MATERIAL S1:**

**MEDLINE (Ovid): 1946 to March Week 3 2016**

**Date searched: 30.3.16**

**Records found: 3198**

1 ((immunochem$ or immuno-chem$ or immunohistochem$ or immuno-histochem$ or immunol$ or immunochromatographic or immuno-chromatographic or immunoassay or immuno assay) adj4 (f?ecal or f?eces or stool or stools)).ti,ab,ot,hw. (910)

2 iFOBT.ti,ab,ot,hw. (83)

3 1 or 2 (932)

4 F?ecal h?emoglobin.ti,ab,ot,hw. (94)

5 H?emoccult.ti,ab,ot,hw. (669)

6 FOBT.ti,ab,ot,hw. (1013)

7 (guaiac$ or gFOBT).ti,ab,ot,hw. (3444)

8 Guaiac/ (319)

9 or/4-8 (4898)

10 (f?ecal or f?eces or stool or stools).ti,ab,ot,hw. (246345)

11 occult blood/ or occult blood.ti,ab,ot,hw. (6307)

12 (test$ or measur$ or screen$ or exam$).ti,ab,ot,hw. (6577832)

13 10 and 11 and 12 (3172)

14 3 or 9 or 13 (7254)

15 exp colorectal neoplasms/ (162552)

16 exp cecal neoplasms/ (4892)

17 ((colorect$ or rectal$ or rectum$ or colon$ or sigma$ or sigmo$ or rectosigm$ or bowel$ or anal or anus) adj3 (cancer$ or neoplas$ or oncolog$ or malignan$ or tumo?r$ or carcinoma$ or adenocarcinoma$ or sarcoma$ or adenom$ or lesion$)).ti,ab,ot,hw. (199488)

18 CRC.ti,ab,ot. (13848)

19 ((cecum or cecal or caecum or caecal or il?eoc?ecal or il?eoc?ecum) adj3 (cancer$ or neoplas$ or oncolog$ or malignan$ or tumo?r$ or carcinoma$ or adenocarcinoma$ or sarcoma$ or adenom$ or lesion$)).ti,ab,ot. (1846)

20 (large intestin$ adj3 (cancer$ or neoplas$ or oncolog$ or malignan$ or tumo?r$ or carcinoma$ or adenocarcinoma$ or sarcoma$ or adenom$ or lesion$)).ti,ab,ot. (1675)

21 (lower intestin$ adj3 (cancer$ or neoplas$ or oncolog$ or malignan$ or tumo?r$ or carcinoma$ or adenocarcinoma$ or sarcoma$ or adenom$ or lesion$)).ti,ab,ot. (26)

22 15 or 16 or 17 or 18 or 19 or 20 or 21 (208172)

23 14 and 22 (3206)

24 (FOB gold$ or FOBgold$).ti,ab. (11)

25 (jack-arc$ or jackarc$ or HM-JACKarc$).ti,ab. (0)

26 (RIDASCREEN$ H?emo$ or RIDASCREEN$ Hapto$).ti,ab. (1)

27 (OC Sensor$ or OC-Sensor$ or OC Pledia$ or OC-Pledia$).ti,ab. (38)

28 or/24-27 (43)

29 23 or 28 (3208)

30 exp animals/ not (exp animals/ and humans/) (4205567)

**31 29 not 30 (3198)**

**Embase (Ovid): 1974 to 2016 March 29**

**Date searched: 30.3.16**

**Records found: 5255**

1 Fecal Immunochemical Test/ [EMTREE candidate term 13.1.16] (135)

2 ((immunochem$ or immuno-chem$ or immunohistochem$ or immuno-histochem$ or immunol$ or immunochromatographic or immuno-chromatographic or immunoassay or immuno assay) adj4 (f?ecal or f?eces or stool or stools)).ti,ab,ot,hw. (1630)

3 iFOBT.ti,ab,ot,hw. (164)

4 1 or 2 or 3 (1672)

5 F?ecal h?emoglobin.ti,ab,ot,hw. (155)

6 H?emoccult.ti,ab,ot,hw. (866)

7 FOBT.ti,ab,ot,hw. (1857)

8 (guaiac$ or gFOBT).ti,ab,ot,hw. (5159)

9 Guaiac/ (696)

10 or/5-9 (7538)

11 (f?ecal or f?eces or stool or stools).ti,ab,ot,hw. (347542)

12 occult blood/ or occult blood.ti,ab,ot,hw. (11184)

13 (test$ or measur$ or screen$ or exam$).ti,ab,ot,hw. (9112793)

14 11 and 12 and 13 (5121)

15 4 or 10 or 14 (11582)

16 exp colon tumor/ (239813)

17 exp rectum tumor/ (182401)

18 exp colon cancer/ (191557)

19 exp rectum cancer/ (148462)

20 ((colorect$ or rectal$ or rectum$ or colon$ or sigma$ or sigmo$ or rectosigm$ or bowel$ or anal or anus) adj3 (cancer$ or neoplas$ or oncolog$ or malignan$ or tumo?r$ or carcinoma$ or adenocarcinoma$ or sarcoma$ or adenom$ or lesion$)).ti,ab,ot,hw. (318031)

21 CRC.ti,ab,ot. (27205)

22 ((cecum or cecal or caecum or caecal or il?eoc?ecal or il?eoc?ecum) adj3 (cancer$ or neoplas$ or oncolog$ or malignan$ or tumo?r$ or carcinoma$ or adenocarcinoma$ or sarcoma$ or adenom$ or lesion$)).ti,ab,ot. (2649)

23 (large intestin$ adj3 (cancer$ or neoplas$ or oncolog$ or malignan$ or tumo?r$ or carcinoma$ or adenocarcinoma$ or sarcoma$ or adenom$ or lesion$)).ti,ab,ot. (1929)

24 (lower intestin$ adj3 (cancer$ or neoplas$ or oncolog$ or malignan$ or tumo?r$ or carcinoma$ or adenocarcinoma$ or sarcoma$ or adenom$ or lesion$)).ti,ab,ot. (29)

25 or/16-24 (329839)

26 15 and 25 (5264)

27 (FOB gold$ or FOBgold$).ti,ab. (27)

28 (jack-arc$ or jackarc$ or HM-JACKarc$).ti,ab. (7)

29 (RIDASCREEN$ H?emo$ or RIDASCREEN$ Hapto$).ti,ab. (2)

30 (OC Sensor$ or OC-Sensor$ or OC Pledia$ or OC-Pledia$).ti,ab. (151)

31 or/27-30 (169)

32 26 or 31 (5274)

33 animal/ (1733374)

34 animal experiment/ (1919080)

35 (rat or rats or mouse or mice or murine or rodent or rodents or hamster or hamsters or pig or pigs or porcine or rabbit or rabbits or animal or animals or dogs or dog or cats or cow or bovine or sheep or ovine or monkey or monkeys).ti,ab,ot,hw. (6178883)

36 or/33-35 (6178883)

37 exp human/ (16972709)

38 human experiment/ (350478)

39 or/37-38 (16974155)

40 36 not (36 and 39) (4862933)

**41 32 not 40 (5255)**
