# Supplementary material for: Faecal immunochemical tests (FIT) can help to rule out colorectal cancer in patients presenting in primary care with lower abdominal symptoms: a systematic review conducted to inform new NICE DG30 diagnostic guidance
Source: BMC Med. 2017 Oct 24;15:189. doi: 10.1186/s12916-017-0944-z (PMC5654140; doi:10.1186/s12916-017-0944-z)
Supplement: Supplementary file 2 — Details of included studies and related publications. (DOCX 18 kb) [file 12916_2017_944_MOESM2_ESM.docx]

**Table S1: DETAILS OF INCLUDED STUDIES AND RELATED PUBLICATIONS**

| ***OC-sensor*** | |
| --- | --- |
| **Primary publication** | **Additional articles** |
| McDonald PJ, Digby J, Innes C, Strachan JA, Carey FA, Steele RJ, et al. Low faecal haemoglobin concentration potentially rules out significant colorectal disease. *Colorectal Dis* 2013;15(3):e151-9. | None |
| Mowat C, Digby J, Strachan JA, Wilson R, Carey FA, Fraser CG, et al. Faecal haemoglobin and faecal calprotectin as indicators of bowel disease in patients presenting to primary care with bowel symptoms. *Gut* 2015:doi:10.1136/gutjnl-2015-309579. | Steele R, Digby J, Strachan J, Mowat C, Lang J, McDonald P, et al. Quantitative FIT as triage for colonoscopy [Presentation]. *WEO Colorectal Cancer Screening Meeting*: Vienna, Austria, 2014. |
| Rodriguez-Alonso L, Rodriguez-Moranta F, Ruiz-Cerulla A, Lobaton T, Arajol C, Binefa G, et al. An urgent referral strategy for symptomatic patients with suspected colorectal cancer based on a quantitative immunochemical faecal occult blood test. *Dig Liver Dis* 2015;47(9):797-804. | None |
| Cubiella J, Salve M, Diaz-Ondina M, Vega P, Alves MT, Iglesias F, et al. Diagnostic accuracy of the faecal immunochemical test for colorectal cancer in symptomatic patients: comparison with NICE and SIGN referral criteria. *Colorectal Dis* 2014;16(8):O273-82. | Diaz Ondina M, Blanco Vila MI, Ceballos Ogando S, Salve Bouzo M, Macia Cortinas P, Cubiella Fernandez J. Clinical or analytical criteria for colorectal cancer (CRC) detection in symptomatic patients? A diagnostic tests study. *Clin Chem Lab Med* 2014;52:S384. |
|  | Cubiella J. Colorectal cancer prediction model in symptomatic patients based on FIT, age and sex [Presentation]. *WEO Colorectal Cancer Screening Meeting*: Barcelona, Spain, 2015. |
|  | Cubiella J, Vega P, Salve M, Diaz-Ondina M, Alves MT, Quintero E, et al. Development and external validation of a faecal immunochemical test-based prediction model for colorectal cancer detection in symptomatic patients. *BMC Med* 2016;14(1):128. |
|  | Cubiella, J. Digby, F. Rodriguez-Moranta, L. Bujanda, P. Vega, M. Diaz-Ondina, L. Rodrıguez-Alonso, J. Guardiola, C. Fraser, on behalf of CriPS. the fas (fecal hemoglobin concentration, age and sex) model: development and external validation of a simple predictive model for colorectal cancer detection in symptomatic patients. United European Gastroenterology Journal 3(5S), A122. United European Gastroenterology Week |
| Terhaar sive Droste JS, Oort FA, van der Hulst RW, van Heukelem HA, Loffeld RJ, van Turenhout ST, et al. Higher fecal immunochemical test cutoff levels: lower positivity rates but still acceptable detection rates for early-stage colorectal cancers. *Cancer Epidemiol Biomarkers Prev* 2011;20(2):272-80. | Oort FA, van Turenhout ST, Coupe VM, van der Hulst RW, Wesdorp EI, Terhaar sive Droste JS, et al. Double sampling of a faecal immunochemical test is not superior to single sampling for detection of colorectal neoplasia: a colonoscopy controlled prospective cohort study. *BMC Cancer* 2011;11:434. |
|  | van Turenhout ST, Oort FA, Coupe VM, Van Der Hulst RW, Wesdorp EC, Larbi IB, et al. Double versus single sampling of fecal immunochemical tests for colorectal cancer screening; added value or added costs? *Gastroenterology* 2010;138(5 Suppl 1):S185. |
|  | van Turenhout ST, Oort FA, van der Hulst RW, Visscher AP, Terhaar sive Droste JS, Scholten P, et al. Prospective cross-sectional study on faecal immunochemical tests: sex specific cut-off values to obtain equal sensitivity for colorectal cancer? *BMC Gastroenterology* 2014;14:217. |
|  | van Turenhout ST, Oort FA, Terhaar sive Droste JS, Coupe VM, van der Hulst RW, Loffeld RJ, et al. Hemorrhoids detected at colonoscopy: an infrequent cause of false-positive fecal immunochemical test results. *Gastrointest Endosc* 2012;76(1):136-43. |
|  | van Turenhout ST, Oort FA, Droste JSTS, Visscher AP, Coupe VM, Van Der Hulst RW, et al. Gender disparities in performance of a fecal immunochemical test for detection of advanced neoplasia. *Gastroenterology* 2011;140(5 Suppl 1):S405-S406. |
|  | Oort FA, Terhaar Sive Droste JS, Van Der Hulst RW, Van Heukelem HA, Loffeld RJ, Wesdorp IC, et al. Colonoscopy-controlled intra-individual comparisons to screen relevant neoplasia: faecal immunochemical test vs. guaiac-based faecal occult blood test. *Aliment Pharmacol Ther* 2010;31(3):432-9. |
|  | van Turenhout ST, van Rossum LG, Oort FA, Laheij RJ, van Rijn AF, Terhaar sive Droste JS, et al. Similar fecal immunochemical test results in screening and referral colorectal cancer. *World J Gastroenterol* 2012;18(38):5397-403. |
|  | Larbi IB, van Turenhout ST, Oort FA, Sive Droste JST, Van Der Hulst RW, Scholten P, et al. FIT in the elderly: performance of a frequently used fecal immunochemical test in subjects 75 of age and older. *Gastroenterology* 2012;142(5 Suppl 1):S773. |
|  | van Turenhout ST, Oort FA, Coupe VM, Van Der Hulst RW, Wesdorp EC, Larbi IB, et al. Comparing three different strategies of double sampling by fecal immunochemical tests for detection of advanced colorectal neoplasm's. *Gastroenterology* 2010;138(5 Suppl 1):S134. |
| ***HM-JACKarc*** | |
| Thomas CL, Tomkins C, Widlak M, Smith S, Arasaradnam R. Can immunochemical tests for faecal haemoglobin and faecal calprotectin be used to risk stratify patients for referral to colonoscopy for suspected colorectal cancer? *Ann Clin Biochem* 2016;53(Suppl 1):38-9. | None |
| Godber IM, Todd LM, Fraser CG, MacDonald LR, Younes HB. Use of a faecal immunochemical test for haemoglobin can aid in the investigation of patients with lower abdominal symptoms. *Clin Chem Lab Med* 2016;54(4):595-602. | Macdonald LR, Smith L, Godber IM, Todd LM, Fraser CG, Downey M, et al. Faecal immunochemical testing for haemoglobin in symptomatic patients can help decide need for colonoscopy. *Gut* 2015;64:A49. |
|  | Godber IM, Todd LM, Fraser CG, Robertson C, Smith L, McDonald L, et al. Can an automated faecal immunochemical test (FIT) determine whether faecal haemoglobin (f-Hb) concentrations can aid in stratifying symptomatic patients referred for colonoscopy. *Clin Chem Lab Med* 2014;52(11):eA268. |
| Auge JM, Fraser CG, Rodriguez C, Roset A, Lopez-Ceron M, Grau J, et al. Clinical utility of one versus two faecal immunochemical test samples in the detection of advanced colorectal neoplasia in symptomatic patients. *Clin Chem Lab Med* 2016;54(1):125-32. | Auge Fradera JM, Roset A, Escudero JM, Foj L, Filella X, Molina R. Clinical utility of HM JACKarc for the detection of colorectal cancer and high-risk adenomas. *Tumor Biol* 2014;35:S15. |
|  | Auge Fradera JM. The performance of FIT to triage symptomatic patients (Clinical evaluation of "HM-JACKarc" analyser) [Presentation]. Barcelona: Hospital Clinic - Barcelona. Biomedical Diagnostic Center, Biochemistry and Molecular Genetics Department, 2015. |
| ***FOB Gold*** | |
| Krivec S, Konda G, Sibli R, Marc J. Assessment of the diagnostic applicability of quantitative immunochemical faecal occult blood tests. *Clin Chem Lab Med* 2011;49:S587. | None |
