# Supplementary material for: Faecal immunochemical tests (FIT) can help to rule out colorectal cancer in patients presenting in primary care with lower abdominal symptoms: a systematic review conducted to inform new NICE DG30 diagnostic guidance
Source: BMC Med. 2017 Oct 24;15:189. doi: 10.1186/s12916-017-0944-z (PMC5654140; doi:10.1186/s12916-017-0944-z)
Supplement: Supplementary file 3 — Details of excluded studies with rationale for exclusion. (DOCX 37 kb) [file 12916_2017_944_MOESM3_ESM.docx]

Table S2: DETAILS OF EXCLUDED STUDIES WITH RATIONALE FOR EXCLUSION

| **Study Details** | **Primary study** | **Population** | **Setting** | **Index Test** | **Reference Standard** | **Outcome** | **Reason for exclusion and comments** |
| --- | --- | --- | --- | --- | --- | --- | --- |
| Abdullah M, Simadibrata M, Syam AF, Wijayadi T, Fauzi A, Santi A, et al. The accuracy of fecal immunochemical test in early detection of colorectal cancer. *J Gastroenterol Hepatol* 2010;25:A138 | Yes | Unclear | Unclear | No | Yes | Yes | No relevant index test  Qualitative FIT. Population unclear, participants undergoing colonoscopy for any indication |
| Allison JE, Fraser CG, Halloran SP, Young GP. Comparing fecal immunochemical tests: improved standardization is needed. *Gastroenterology* 2012;142(3):422-4 | No |  |  |  |  |  | Not primary research |
| Banaszkiewicz Z, Jawien A, Jarmocik P, Tojek K, Jankowski M, Switonski M. [Evaluation of usefulness of faecal occult blood test. Prospective screening study in patients with colorectal neoplasia]. *Pol Merkuriusz Lek* 2004;17(102):579-82 | Yes | Unclear | Unclear | No | Unclear | Unclear | No relevant index test  Polish language publication, qualitative FIT |
| Bjerregaard NC, Tottrup A, Sorensen HT, Laurberg S. Detection of colorectal cancer in symptomatic outpatients without visible rectal bleeding: validity of the fecal occult blood test. *Clin Epidemiol* 2009;1:119-24 | Yes | Yes | Yes | No | Yes | Yes | No relevant index test  gFOBT only |
| Bonfrate L, Ruggiero V, Dambrosio P, Castorani L, De Bari O, Larizza M, et al. Double vs. standard fecal occult blood testing (FOBT) in patients with alarm symptoms of colorectal cancer (CRC). *Eur J Clin Invest* 2013;43:71 | Yes | Yes | Unclear | No | Yes | Yes | No relevant index test  Qualitative FIT |
| Carroll M, Piggott C, Pearson S, Seaman HE, Bruce H, Halloran SP. An evaluation of quantitative faecal immunochemical tests for haemoglobin [Poster]. *British Society of Gastroenterology Annual Meeting*. Manchester, 2014: 89 | Yes | No | Unclear | Yes | No | No | Not a symptomatic population  Spiked samples, technical performance only |
| Castro I, Estevez P, Cubiella J, Hernandez V, Gonzalez-Mao C, Rivera C, et al. Diagnostic performance of fecal immunochemical test and sigmoidoscopy for advanced right-sided colorectal neoplasms. *Dig Dis Sci* 2015;60(5):1424-32 | Yes | No | Unclear | Yes | Yes | Yes | Not a symptomatic population  Average risk cohort of asymptomatic men |
| Chen JG, Cai J, Wu HL, Xu H, Zhang YX, Chen C, et al. Colorectal cancer screening: comparison of transferrin and immuno fecal occult blood test. *World J Gastroenterol* 2012;18(21):2682-8 | Yes | No | Unclear | No | Yes | Yes | Not a symptomatic population  Mixed population, symptomatic and asymptomatic surveillance. Qualitative FIT |
| Chiang TH, Lee YC, Tu CH, Chiu HM, Wu MS. Immunochemical FOBT was accurate for detecting colorectal cancer but less so for other GI lesions. *Ann Intern Med* 2012;156(4):JC2-JC10 | Yes | No | Unclear | No | Yes | Yes | Not a symptomatic population  Screening with qualitative FIT |
| Chiu HM, Wu MS, Lee YC, Liao WC, Wang HP, Lin JT. Fecal immunochemical test has lower performance in detecting early and proximal advanced colorectal neoplasm. *Gastroenterology* 2012;142(5 Suppl 1):S145 | Yes | No | No | No | Yes | Yes | Not a symptomatic population  Screening population |
| Cole SR, Upton J, Lane JM, Young GP. A faecal immunochemical test for haemoglobin using a single stool sample is effective for detecting significant colorectal neoplasia. *J Gastroenterol Hepatol* 2009;24:A239 | Yes | Unclear | Unclear | No | Yes | Yes | No relevant index test  Unclear population, all patients scheduled for colonoscopy. No relevant FIT |
| Crouse AL, De Koning L, Sadrzadeh SM, Naugler C. Sensitivity and specificity of community fecal immunotesting screening for colorectal carcinoma in a high-risk Canadian population. *Arch Pathol Lab Med* 2015;139(11):1441-5 | Yes | No | Unclear | Yes | Yes | Yes | Not a symptomatic population  Screening population |
| Dutta AK, Alagammai P, Chowdhury SD, Chacko A. Preprocedure haemoglobin and fecal occult blood testing for optimising colonoscopy rates in a resource limited setting for diagnosing colonic malignancy. *Gastrointest Endosc* 2012;75(4 Suppl 1):AB325-AB326 | Yes | No | Unclear | No | Yes | No | Not a symptomatic population  gFOBT only, test performance only reported for combined gFOBT and anaemia |
| Fenocchi E, Gaggero P, Rondan M, Lopez-Alvarenga JC, Sobrino-Cossio S, Lambert N, et al. Usefulness of the fecal immunochemical test in the detection of advanced adenomas in subjects at average risk for colorectal cancer. *Endoscopia* 2015;27(2):64-68 | Yes | Yes | Unclear | Yes | Yes | No | No relevant outcomes  Separate data for symptomatic and asymptomatic patients. Reported outcome was mean FIT level for different adenoma sizes |
| Gillberg A, Ericsson E, Granstrom F, Olsson LI. A population-based audit of the clinical use of faecal occult blood testing in primary care for colorectal cancer. *Colorectal Dis* 2012;14(9):e539-46 | Yes | Unclear | Unclear | No | No | Yes | No relevant index test  Retrospective study, symptoms data only collected for those patients with a diagnosis of CRC |
| Gopalswamy N, Stelling HP, Markert RJ, Maimon HN, Wahlen SD, Haddy RI. A comparative study of eight fecal occult blood tests and HemoQuant in patients in whom colonoscopy is indicated. *Arch Fam Med* 1994;3(12):1043-8 | Yes | No | Unclear | No | Yes | Yes | Not a symptomatic population  Mixed population, symptomatic, asymptomatic high risk and surveillance. No relevant FIT (gFOBT only) |
| Greenberg PD, Bertario L, Gnauck R, Kronborg O, Hardcastle JD, Epstein MS, et al. A prospective multicenter evaluation of new fecal occult blood tests in patients undergoing colonoscopy. *Am J Gastroenterol* 2000;95(5):1331-8 | Yes | No | Unclear | No | Yes | Yes | Not a symptomatic population  Mixed population, symptomatic and asymptomatic screening, history of CRC or polyps, or family history (30% symptomatic). No relevant FIT technology |
| Haug U, Kuntz KM, Knudsen AB, Hundt S, Brenner H. Sensitivity of immunochemical faecal occult blood testing for detecting left- vs right-sided colorectal neoplasia. *Br J Cancer* 2011;104(11):1779-85 | Yes | No | Unclear | Yes | Yes | Yes | Not a symptomatic population  Average risk screening population |
| Hogberg C, Karling P, Rutegard J, Lilja M, Ljung T. Immunochemical faecal occult blood tests in primary care and the risk of delay in the diagnosis of colorectal cancer. *Scand J Prim Health Care* 2013;31(4):209-14 | Yes | No | Unclear | Unclear | N/A | No | Patients with an established cancer diagnosis  Retrospective study of cancer patients who had received qualitative FIT, reports sensitivity of FIT and delay to diagnosis following a negative FIT |
| Iwase N, Oya M, Yanagida T, Hirayasu Y, Ishii Y, Kubota T, et al. [Immunological fecal occult blood test in patients with anal diseases]. *Nihon Daicho Komonbyo Gakkai Zasshi* 1995;48(9):1065-1069 | Yes | Yes | Unclear | No | Yes | No | No relevant index test  Japanese language publication, gFOBT only. Only sensitivity data reported |
| Jin P, Wu Z, Meng M, Wang X, Gong L, Yu D, et al. Combined fecal transferrin test and immuno fecal occult blood test for detecting colorectal cancer and advanced adenoma in asymptomatic and symptomatic populations. *J Cancer Sci Ther* 2012;4(8):243-248 | Yes | Yes | Unclear | No | Yes | No | No relevant index test  Qualitative FIT, no accuracy data |
| Kalimutho M, Del Vecchio Blanco G, Cretella M, Mannisi E, Sileri P, Formosa A, et al. A simplified, non-invasive fecal-based DNA integrity assay and iFOBT for colorectal cancer detection. *Int J Colorectal Dis* 2011;26(5):583-92 | Yes | Yes | Yes | No | Yes | Yes | No relevant index test  Qualitative FIT |
| Kaul A, Shah A, Magill FH, Hawkins SA, Skaife P. Immunological faecal occult blood testing: a discriminatory test to identify colorectal cancer in symptomatic patients. *Int J Surg* 2013;11(4):329-31 | Yes | Yes | Yes | No | Yes | Yes | No relevant index test  Qualitative FIT |
| Kennell M, Antle S, Hammond M, Stone S, Mahar D, Randell E. Evaluation of the analytical and diagnostic performance of the iFOBT NS-Plus system for use in a province-wide colorectal cancer screening program. *Clin Biochem* 2012;45(13-14):1116 | Yes | Unclear | Unclear | No | Yes | Yes | Not a symptomatic population  No relevant index test |
| Ko CW, Dominitz JA, Nguyen TD. Fecal occult blood testing in a general medical clinic: comparison between guaiac-based and immunochemical-based tests. *Am J Med* 2003;115(2):111-4 | Yes | No | Unclear | No | No | No | Not a symptomatic population  Screening with qualitative FIT |
| Kovarova JT, Zavoral M, Zima T, Zak A, Kocna P, Kohout P, et al. Improvements in colorectal cancer screening programmes - quantitative immunochemical faecal occult blood testing - how to set the cut-off for a particular population. *Biomed Pap Med Fac Univ Palacky Olomouc Czech Repub* 2012;156(2):143-50 | Yes | No | Unclear | Yes | Yes | Yes | Not a symptomatic population  Mixed population, symptomatic and asymptomatic, previous CRC, family history (32% symptomatic) – **authors contacted, reply received stating that subgroup data are not available** |
| Leicester RJ, Lightfoot A, Millar J, Colin-Jones DG, Hunt RH. Accuracy and value of the Hemoccult test in symptomatic patients. *BMJ* 1983;286(6366):673-4 | Yes | Yes | Yes | No | No | Yes | No relevant index test  gFOBT only. Reference standard proctosigmoidoscopy and double contrast barium enema (colonoscopy in patients with normal findings on these tests who had positive FOBT results) |
| Leodolter A, Zielinski D, Vieth M, Labenz J. Comparison of different immunological fobts for colorectal cancer screening: wide range of sensitivity between different rapid tests. *Gastroenterology* 2010;138(5 Suppl 1):S159 | Yes | No | Unclear | Yes | Yes | Yes | Not a symptomatic population  Mixed population, symptomatic and asymptomatic (approximately 66% symptomatic) – **authors contacted, no reply received** |
| Levi Z, Hazazi R, Rozen P, Vilkin A, Waked A, Niv Y. A quantitative immunochemical faecal occult blood test is more efficient for detecting significant colorectal neoplasia than a sensitive guaiac test. *Aliment Pharmacol Ther* 2006;23(9):1359-64 | Yes | No | No | Yes | Yes | Yes | Not a symptomatic population  Asymptomatic screening |
| Levi Z, Rozen P, Hazazi R, Vilkin A, Waked A, Maoz E, et al. A quantitative immunochemical fecal occult blood test for colorectal neoplasia. *Ann Intern Med* 2007;146(4):244-55 | Yes | No | Yes | Yes | Yes | Yes | Not a symptomatic population  Mixed population symptomatic and asymptomatic screening or high risk (47% symptomatic) – **authors contacted, no reply received^*^** |
| Levi Z, Rozen P, Hazazi R, Vilkin A, Waked A, Maoz E, et al. Sensitivity, but not specificity, of a quantitative immunochemical fecal occult blood test for neoplasia is slightly increased by the use of low-dose aspirin, NSAIDs, and anticoagulants. *Am J Gastroenterol* 2009;104(4):933-8 | Yes | No | Unclear | Yes | Yes | Yes | Not a symptomatic population  Mixed population, increased risk and mildly symptomatic, proportions not specified – **authors contacted, no reply received^*^** |
| Levy BT, Bay C, Xu Y, Daly JM, Bergus G, Dunkelberg J, et al. Test characteristics of faecal immunochemical tests (FIT) compared with optical colonoscopy. *J Med Screen* 2014;21(3):133-43 | Yes | No | Unclear | No | Yes | Yes | Not a symptomatic population  Mixed population (symptomatic, screening or surveillance), proportion not reported. No relevant FIT technology |
| Luthgens K, Maier A, Kampert I, Sieg A, Schmidt-Gayk H. Hemoglobin-haptoglobin-complex: a highly sensitive assay for the detection of fecal occult blood. *Clinical Laboratory* 1998;44(7-8):543-551 | Yes | Unclear | Unclear | No | Yes | Yes | No relevant index test  Participants from a gastroenterological practice, unclear if symptomatic. Study is of a development version of the test not that which is currently marketed |
| McDonald R, Tomlins A, Smith S, Harmston C. Outcomes of faecal occult blood tests requested outside the UK National Bowel Cancer Screening Programme. *J Clin Pathol* 2013;66(4):330-4 | Yes | Unclear | Unclear | No | N/A | No | No relevant index test  Unclear population, survey of testing requests before and after introduction of screening |
| Miyoshi H, Oka M, Sugi K, Saitoh O, Katsu K, Uchida K. Accuracy of detection of colorectal neoplasia using an immunochemical occult blood test in symptomatic referred patients: comparison of retrospective and prospective studies. *Intern Med* 2000;39(9):701-6 | Yes | No | Unclear | No | Yes | Yes | Not a symptomatic population  Qualitative FIT |
| Narula N, Ulic D, Al-Dabbagh R, Ibrahim A, Mansour M, Balion C, et al. Fecal occult blood testing as a diagnostic test in symptomatic patients is not useful: a retrospective chart review. *Can J Gastroenterol Hepatol* 2014;28(8):421-6 | Yes | No | No | No | Yes | Yes | Not a symptomatic population  Hospital in-patients, not all symptomatic, type of FOB test not reported |
| Niv Y, Sperber AD. Sensitivity, specificity, and predictive value of fecal occult blood testing (Hemoccult II) for colorectal neoplasia in symptomatic patients: a prospective study with total colonoscopy. *Am J Gastroenterol* 1995;90(11):1974-7 | Yes | Yes | Yes | No | Yes | Yes | No relevant index test  gFOBT only |
| Ogawa M, Iso A, Ootsuka H, Shimizu S, Aoki Y, Tada M, et al. [Clinical evaluation of a new immunological fecal occult blood test]. *Ther Res* 1989;10(2):275-282 | Yes | No | Unclear | No | No | Yes | No relevant index test  Japanese language publication, qualitative FIT, case-control study |
| Oono Y, Iriguchi Y, Doi Y, Tomino Y, Kishi D, Oda J, et al. A retrospective study of immunochemical fecal occult blood testing for colorectal cancer detection. *Clin Chim Acta* 2010;411(11-12):802-5 | Yes | Yes | Yes | No | Yes | Yes | No relevant index test  No relevant FIT technology |
| Oort FA, Droste JSTS, Van Der Hulst RW, Van Heukelem H, Loffeld RJ, Wesdorp EC, et al. Flat colonic neoplasia are left undetected by fecal immunochemical tests (FIT) and will be missed in colorectal cancer screening. *Gastroenterology* 2009;136(5 Suppl 1):A113 | Yes | Unclear | Unclear | Yes | Yes | No | No relevant outcomes  Unclear population, all patients scheduled for colonoscopy. No accuracy data. |
| Ou CH, Kuo FC, Hsu WH, Lu CY, Yu FJ, Kuo CH, et al. Comparison of the performance of guaiac-based and two immunochemical fecal occult blood tests for identifying advanced colorectal neoplasia in Taiwan. *J Dig Dis* 2013;14(9):474-83 | Yes | No | Unclear | Yes | Yes | Yes | Not a symptomatic population  Mixed population (history of CRC or polyp, family history, symptomatic, asymptomatic screening) proportions not reported – **authors contacted, no reply received** |
| Parente FR, Marino B, Perna F, Saracino IM, Zullo A, Hassan C, et al. Multiple faecal tests (colon panel) for the detection of colon cancer: a new strategy for appropriate prioritization of screening referrals? Preliminary experience in Italy. *Gastroenterology* 2010;138(5 Suppl 1):S192. | Yes | Yes | Unclear | No | Yes | Yes | No relevant index test – HM-JACK (no longer available in Europe) not HM-JACKarc |
| Parente F, Marino B, Perna F, Saracino I, Zullo A, Hassan C, et al. Multiple faecal tests (colon panel) for the detection of colon cancer: a new strategy for appropriate prioritization of screening referrals? Preliminary experience in Italy. *Dig Liver Dis* 2010;42:S86. | Yes | Yes | Unclear | No | Yes | Yes | No relevant index test – HM-JACK (no longer available in Europe) not HM-JACKarc |
| Parente F, Marino B, Ilardo A, Fracasso P, Zullo A, Hassan C, et al. A combination of faecal tests for the detection of colon cancer: a new strategy for an appropriate selection of referrals to colonoscopy? A prospective multicentre Italian study. *Eur J Gastroenterol Hepatol* 2012;24(10):1145-52. | Yes | Yes | Unclear | No | Yes | Yes | No relevant index test – HM-JACK (no longer available in Europe) not HM-JACKarc |
| Piper MA. *Immunochemical versus guaiac fecal occult blood tests*. Chicago, IL: Blue Cross and Blue Shield Association, Technology Evaluation Center, 2004 | No |  |  |  |  |  | Not primary research  Provisional DARE abstract, with no publication details |
| Rae AJ, Cleator IGM. The two-tier fecal occult blood test: cost effective screening. *Can J Gastroenterol* 1994;8(6):362-368 | Yes | No | Unclear | No | No | No | Not a symptomatic population  Mixed population, symptomatic and asymptomatic screening and surveillance, gFOBT only |
| Rao S, Forbes G, Venugopal K. High yield for advanced colorectal neoplasia (carcinoma and advanced adenoma) detection with community based faecal immunochemical testing. *J Gastroenterol Hepatol* 2014;29:133-134 | Yes | No | Unclear | Unclear | Yes | No | Not a symptomatic population  Patients referred from primary care because of a positive FIT. FIT method not specified |
| Rodriguez-Moranta F, Ariza X, Berrozpe A, Vazquez X, Binefa G, Navarro M, et al. Comparative study of guaiac and quantitative immunochemical fecal occult blood test for colorectal neoplasia. Preliminary results. *Gastroenterology* 2009;136(5 Suppl 1):A623 | Yes | No | Unclear | No | Yes | Yes | Not a symptomatic population  Mixed population, symptomatic and asymptomatic screening, or surveillance (66% symptomatic). Un-specified FIT – **authors contacted, no reply received** |
| Rozen P, Levi Z, Hazazi R, Waked A, Vilkin A, Maoz E, et al. Quantitative colonoscopic evaluation of relative efficiencies of a quantified immunochemical fecal occult blood test and a sensitive guaiac test for detecting significant colorectal neoplasms. *Gastroenterology* 2009;136(5 Suppl 1):A113 | Yes | No | Unclear | Yes | Yes | Yes | Not a symptomatic population  Unclear population, 'consecutive ambulatory colonoscopy patients, some above average risk'– **authors contacted, no reply received^*^** |
| Rozen P, Levi Z, Hazazi R, Waked A, Vilkin A, Maoz E, et al. Identification of colorectal adenomas by a quantitative immunochemical faecal occult blood screening test depends on adenoma characteristics, development threshold used and number of tests performed. *Aliment Pharmacol Ther* 2009;29(8):906-17 | Yes | No | Unclear | Yes | Yes | Yes | Not a symptomatic population  Mixed population, symptomatic and high risk asymptomatic (59% symptomatic) – **authors contacted, no reply received^*^** |
| Rozen P, Levi Z, Hazazi R, Waked A, Vilkin A, Maoz E, et al. Colonoscopic evaluation of a quantitative immunochemical fecal occult blood test to determine its optimal clinical use. *Gastroenterology* 2009;136(5 Suppl 1):A624 | Yes | No | Yes | Yes | Yes | Yes | Not a symptomatic population  Unclear population, 'consecutive ambulatory colonoscopy patients, some above average risk'– **authors contacted, no reply received^*^** |
| Rozen P, Levi Z, Hazazi R, Waked A, Vilkin A, Maoz E, et al. Quantitative colonoscopic evaluation of relative efficiencies of an immunochemical faecal occult blood test and a sensitive guaiac test for detecting significant colorectal neoplasms. *Aliment Pharmacol Ther* 2009;29(4):450-7 | Yes | No | Yes | Yes | Yes | Yes | Not a symptomatic population  Mixed population, symptomatic and asymptomatic screening or high risk (23% symptomatic) – **authors contacted, no reply received^*^** |
| Rozen P, Comaneshter D, Levi Z, Hazazi R, Vilkin A, Maoz E, et al. Cumulative evaluation of a quantitative immunochemical fecal occult blood test to determine its optimal clinical use. *Cancer* 2010;116(9):2115-25 | Yes | No | No | Yes | Yes | Yes | Not a symptomatic population  Screening asymptomatic |
| Sailer M. [A quantitative immunological fecal occult blood test in colorectal neoplasia]. *Coloproctology* 2010;32(1):68-70 | No |  |  |  |  |  | Not primary research  Journal club, screening |
| Sailer M. [The sensitivity and specificity of guaiac and immunochemical fecal occult blood tests for the detection of advanced colonic adenomas and cancer]. *Coloproctology* 2013;35(2):148-150 | No |  |  |  |  |  | Not primary research  Journal club, screening |
| Sieg A, Scheida M, John MR, Hertel A, Schroter M, Luthgens K, et al. Validity of new immunological human fecal hemoglobin and albumin tests in detecting colorectal neoplasms - an endoscopy-controlled study. *Z Gastroenterol* 1998;36(6):485-90 | Yes | Yes | Yes | No | Yes | Yes | No relevant index test  Early development paper (not a commercially available test) |
| Shaw AG, Lund JN, Longman C, Tierney GM, Goddard AF. The misuse of the faecal occult blood test under the lower gastrointestinal two week wait rule. *Colorectal Dis* 2009;11(1):94-6 | Yes | Yes | Yes | No | N/A | No | No relevant index test  Survey of gFOBT testing prior to referral (no test performance data) |
| Symonds EL, Young GP, Osborne JM, Cole SR, Gopalsamy G, Gaur S, et al. Detection of colorectal neoplasia: comparison of a methylated two-gene (BCAT1-IKZF1) blood test with a faecal immunochemical test. *J Gastroenterol Hepatol* 2015;30:83 | Yes | No | Unclear | Yes | Yes | Yes | Not a symptomatic population  Mixed population: States 'scheduled for colonoscopy for any reason,' but the objective describes test evaluation in 'a patient population exhibiting the full spectrum of pathology encountered in the colon/rectum’– **authors contacted, no reply received** |
| Tate JJ, Northway J, Royle GT, Taylor I. Faecal occult blood testing in symptomatic patients: comparison of three tests. *Br J Surg* 1990;77(5):523-6 | Yes | Yes | Yes | No | No | Yes | No relevant index test  gFOBT only. Patients referred for double contrast barium enema (reference standard), assumed by the authors to be symptomatic |
| Thomas WM, Hardcastle JD, Jackson J, Pye G. Chemical and immunological testing for faecel occult blood: a comparison of two tests in symptomatic patients. *Br J Cancer* 1992;65(4):618-20 | Yes | Yes | Yes | No | Yes | No | No relevant index test  Only sensitivity data reported |
| Tibble J, Sigthorsson G, Foster R, Sherwood R, Fagerhol M, Bjarnason I. Faecal calprotectin and faecal occult blood tests in the diagnosis of colorectal carcinoma and adenoma. *Gut* 2001;49(3):402-8 | Yes | No | Unclear | No | Yes | Yes | Not a symptomatic population  Mixed population, healthy controls, known CRC and referred patients (symptomatic and polyp surveillance, proportions not specified). No relevant FIT technology |
| Tsumuraya M, Noda A, Tsubura S, Sugimoto K, Minowa M, Seki T, et al. [Comparative clinical study of 'Monohem' and four reagents for faecal occult blood test]. *Ther Res* 1989;10(Suppl 1):87-95 | Yes | No | Unclear | No | No | Yes | No relevant index test  Japanese language publication, qualitative FIT, case-control study |
| University of Aarhus. A Trial of the Implementation of iFOBT in General Practice. NCT02308384 In: International Clinical Trials Registry Platform [Internet]. Geneva: World Health Organization. 2014 [accessed 9.3.16]. Available from: http://apps.who.int/trialsearch/Trial2.aspx?TrialID=NCT02308384 | Yes | Yes | Yes | Unclear | N/A | No | No relevant outcomes  Ongoing study, behavioural intervention for general practitioners |
| University of Malaya. Quantitative Versus Qualitative Fecal Immunochemical Tests (FIT) to Prioritize Urgency of Colonoscopy Referral. NCT02037646 In: International Clinical Trials Registry Platform [Internet]. Geneva: World Health Organization. 2014 [accessed 9.3.16]. Available from: http://apps.who.int/trialsearch/Trial2.aspx?TrialID=NCT02037646 | Yes | No | No | Unclear | Yes | Yes | Not a symptomatic population  Screening study, index test and comparator unclear |
| Uppsala University. Quantitative Immunochemical Fecal Occult Blood Test in Symptomatic Patients. NCT02491593 In: ClinicalTrials.gov [Internet]. Bethesda (MD): National Library of Medicine (US). 2015 [accessed 9.3.16]. Available from: https://ClinicalTrials.gov/show/NCT02491593 | Yes | Yes | Yes | Unclear | Unclear | Yes | No data available  Ongoing study, potentially relevant. Unclear intervention, quantitative FIT. Un-specified reference standard |
| Van Turenhout ST, Van Rossum LG, Oort FA, Laheij RJ, Van Rijn AF, Fockens P, et al. Differences in FIT results between screening and referred colorectal cancer patients are explained by differences in tissue tumor stage. *Gastroenterology* 2010;138(5 Suppl 1):S185 | Yes | No | Unclear | Yes | Yes | No | Not a symptomatic population  Comparison of stages of CRC between screening detected and symptomatic patients |
| Viana Freitas BR, Kibune Nagasako C, Pavan CR, Silva Lorena SL, Guerrazzi F, Saddy Rodrigues Coy C, et al. Immunochemical fecal occult blood test for detection of advanced colonic adenomas and colorectal cancer: comparison with colonoscopy results. *Gastroenterol Res Pract* 2013;2013:384561 | Yes | No | No | No | Yes | Yes | Not a symptomatic population  Mixed population, symptomatic and asymptomatic high risk, or surveillance. Qualitative FIT |
| Vilkin A, Rozen P, Levi Z, Waked A, Maoz E, Birkenfeld S, et al. Performance characteristics and evaluation of an automated-developed and quantitative, immunochemical, fecal occult blood screening test. *Am J Gastroenterol* 2005;100(11):2519-25 | Yes | No | Yes | Yes | Yes | Yes | Not a symptomatic population  Mixed population, symptomatic and asymptomatic high risk (approximately 73% symptomatic) – **authors contacted, no reply received^*^** |
| Vogel T, Driemel C, Hauser A, Hansmann A, Lange S, Jonas M, et al. [Comparison of different stool tests for the detection of cancer of the colon]. *Dtsch Med Wochenschr* 2005;130(14):872-7 | Yes | No | No | No | Yes | Yes | Not a symptomatic population  Includes health control participants. No relevant FIT technology |
| Wakamura K, Kudo SE, Ikehara N, Mori Y, Hayashi S, Takeda K, et al. A prospective evaluation using the colonoscope of the fecal occult blood test-negative colorectal neoplasms in a referral hospital. *Gastrointest Endosc* 2012;75(4 Suppl 1):AB343-AB344 | Yes | No | Unclear | No | Yes | Yes | Not a symptomatic population  Mixed population (approximately 25% symptomatic). Unspecified FIT method – **authors contacted, reply received: No relevant FIT technology** |
| de Wijkerslooth TR, Stoop EM, Bossuyt PM, Meijer GA, van Ballegooijen M, van Roon AH, et al. Immunochemical fecal occult blood testing is equally sensitive for proximal and distal advanced neoplasia. *Am J Gastroenterol* 2012;107(10):1570-8 | Yes | No | Unclear | Yes | Yes | Yes | Not a symptomatic population  Asymptomatic screening |
| Williams JA, Hunter R, Coles ME, Thomas DW, Huber TW. An assessment of an immunochemical test for human haemoglobin in the detection of colonic polyps. *Aust N Z J Surg* 1985;55(5):485-8 | Yes | No | Unclear | Unclear | Yes | No | Not a symptomatic population  Mixed population symptomatic and asymptomatic, or history of CRC (proportions not reported). FIT type not reported |
| Wong BC, Wong WM, Cheung KL, Tong TS, Rozen P, Young GP, et al. A sensitive guaiac faecal occult blood test is less useful than an immunochemical test for colorectal cancer screening in a Chinese population. *Aliment Pharmacol Ther* 2003;18(9):941-6 | Yes | No | Unclear | No | Yes | No | Not a symptomatic population  Mixed population, symptomatic and asymptomatic surveillance (46% symptomatic). FlexSure OBT & Hemoccult SENSA, no relevant FIT technology. Only sensitivity data reported |
| Wong WM, Lam SK, Cheung KL, Tong TS, Rozen P, Young GP, et al. Evaluation of an automated immunochemical fecal occult blood test for colorectal neoplasia detection in a Chinese population. *Cancer* 2003;97(10):2420-4 | Yes | No | No | No | Yes | No | Not a symptomatic population  Mixed population, symptomatic, polyp surveillance, history of CRC, or family history (37% symptomatic). No relevant FIT technology. Only sensitivity data reported |
| Woo HY, Mok RS, Park YN, Park DI, Sung IK, Sohn CI, et al. A prospective study of a new immunochemical fecal occult blood test in Korean patients referred for colonoscopy. *Clin Biochem* 2005;38(4):395-9 | Yes | No | Unclear | Yes | Yes | Yes | Not a symptomatic population  Mixed population, symptomatic and asymptomatic or history of CRC (60% symptomatic) – **authors contacted, no reply received** |
| Wu D, Luo HQ, Zhou WX, Qian JM, Li JN. The performance of three-sample qualitative immunochemical fecal test to detect colorectal adenoma and cancer in gastrointestinal outpatients: an observational study. *PLoS One* 2014;9(9):e106648 | Yes | No | No | No | Yes | Yes | Not a symptomatic population  Mixed population, symptomatic and asymptomatic history of CRC or polyp. Qualitative FIT |
| Yeasmin F, Ali MA, Rahman MA, Sultana T, Rahman MQ, Ahmed AN. A comparative study of chemical and immunological method of fecal occult blood test in the diagnosis of occult lower gastrointestinal bleeding. *Bangladesh Med Res Counc Bull* 2013;39(2):52-6 | Yes | Unclear | Unclear | Unclear | Yes | No | No relevant outcomes  Only patients who were positive on either FIT or gFOBT received colonoscopy; the sensitivity and specificity of FIT and gFOBT individually were then assessed against colonoscopy in this pre-selected sample. Unclear whether participants were symptomatic, FIT unspecified |

^*^Rozen P is now deceased; co-authors were contacted
