# Supplementary material for: Faecal immunochemical tests (FIT) can help to rule out colorectal cancer in patients presenting in primary care with lower abdominal symptoms: a systematic review conducted to inform new NICE DG30 diagnostic guidance
Source: BMC Med. 2017 Oct 24;15:189. doi: 10.1186/s12916-017-0944-z (PMC5654140; doi:10.1186/s12916-017-0944-z)
Supplement: Supplementary file 4 — Data extraction tables. (DOCX 33 kb) [file 12916_2017_944_MOESM4_ESM.docx]

**TABLE S3: DATA EXTRACTION TABLES**

**Study and participant details**

| **Study Details** | **Selection criteria** | **Participant details** | **Test Manufacturer** |
| --- | --- | --- | --- |
| **Auge 2016^33^**  **Related publications:** Auge 2014^42^; Auge 2015^48^  **Country:** Spain  **Funding:** Menarini Diagnósticos, S.A. provided instruments, reagents and technical support  **Recruitment:** Dec-13 to Mar-14  **Study design:** Diagnostic Cohort | **Inclusion criteria:** Adult patients, attending a hospital outpatient clinic for the investigation of lower abdominal symptoms or polyp surveillance  **Exclusion criteria:** Patients undergoing CRC screening; history of GI bleeding; active rectal bleeding; menstruation; haematuria; known ulcerative colitis  **Study Setting:** Outpatient clinic  **Point in care pathway at which the test is given:** Testing was requested in a secondary care outpatient clinic | **Total Number tested:** 208  **Median age (range):** 63 (22 - 86) years  **Male (%):** 44.2  **Altered bowel habit (%):** NR  **Rectal bleeding (%):** NR  **Diarrhoea (%):** NR  **Abdominal pain (%):** NR  **Anaemia (un-specified) (%):** NR  **Weight loss (%):** NR  **Palpable mass (%):** NR  **Constipation (%):** NR  **Anaemia (iron deficiency) (%):** NR  **NICE criteria for urgent referral (%):** NR  **SIGN criteria for urgent referral (%):** NR  **Clinical suspicion of CRC (%):** NR | Kyowa Medex, Japan |
| **Cubiella 2014 (COLONPREDICT)^30^**  **Related publications:** Diaz Ondina 2014^37^; Cubiella 2015^52^  **Country:** Spain  **Funding:** Instituto de Salud Carlos III, Madrid, Spain (PI11/00094)  **Recruitment:** Apr-12 to Nov-12  **Study design:** Diagnostic Cohort | **Inclusion criteria:** Adult outpatients, with GI symptoms, referred from primary and secondary healthcare  **Exclusion criteria:** Pregnancy; undergoing colonoscopy for surveillance following previous history of colonic disease; requiring hospital admission; symptoms had ceased within 3 months before the evaluation  **Study Setting:** Mixed  **Point in care pathway at which the test is given:** Testing was requested after referral to the gastroenterology outpatient clinic and before colonoscopy  **Comments:** Data were also presented for the diagnostic performance of individual signs and symptoms and for NICE and SIGN referral criteria. Patients were recruited from primary care and outpatient settings | **Total Number tested:** 787 (Completed protocol)  **Median age (range):** 67 (22 - 91) years  **Male (%):** 51.1  **Altered bowel habit (%):** 56.2  **Rectal bleeding (%):** 64  **Diarrhoea (%):** NR  **Abdominal pain (%):** 44.5  **Anaemia (un-specified) (%):** NR  **Weight loss (%):** 21.2  **Palpable mass (%):** 2.3  **Constipation (%):** 29.2  **Anaemia (iron deficiency) (%):** NR  **NICE criteria for urgent referral (%):** 38.1  **SIGN criteria for urgent referral (%):** 60.4  **Clinical suspicion of CRC (%):** NR | Eiken Chemical Co., Tokyo, Japan |
| **Godber 2016^32^**  **Related publications:** Macdonald 2015^47^; Godber 2014^41^  **Country:** Scotland  **Funding:** NHS Lanarkshire; analyser, training and consumables were supplied by kyowa-Medex Co., Ltd., Tokyo, japan  **Recruitment:** Jun-13 to Dec-13  **Study design:** Diagnostic Cohort | **Inclusion criteria:** Patients (>16 years old) who had been referred from primary care for endoscopic examination of the lower gastrointestinal tract. Most common resons for referral included: altered bowel habit, constipation or diarrhoea, abdominal pain, fresh rectal bleeding, follow-up of previous disease, anaemia, weight loss and abnormal computerised tomography findings.  **Exclusion criteria:** Referred for investigation of a positive test result from the Scottish Bowel Screening Programme  **Study Setting:** outpatient clinic  **Point in care pathway at which the test is given:** Testing was requested after referral for endoscopy. | **Total Number tested:** 507 (Tested for FIT and colonoscopy attempted (484 analysed)  **Median age (range):** 59 (16 - 89) years  **Male (%):** 42.6  **Altered bowel habit (%):** 37.9  **Rectal bleeding (%):** 15.2  **Diarrhoea (%):** NR  **Abdominal pain (%):** 17.9  **Anaemia (un-specified) (%):** 4.5  **Weight loss (%):** 1.6  **Palpable mass (%):** NR  **Constipation (%):** NR  **Anaemia (iron deficiency) (%):** NR  **NICE criteria for urgent referral (%):** NR  **SIGN criteria for urgent referral (%):** NR  **Clinical suspicion of CRC (%):** NR | Kyowa-Medex, japan |
| **Krivec 2011^29^**  **Related publications:**  None  **Country:** Slovenia  **Funding:** NR  **Recruitment:** NR  **Study design:** Diagnostic Cohort | **Inclusion criteria:** Adult symptomatic patients, undergoing endoscopic and/or histological examinations  **Exclusion criteria:** NR  **Study Setting:** unclear  **Point in care pathway at which the test is given:** Unclear (patients scheduled for endoscopy)  **Comments:**  Abstract only | **Median age (range):** NR  **Male (%):** NR  **Altered bowel habit (%):** NR  **Rectal bleeding (%):** NR  **Diarrhoea (%):** NR  **Abdominal pain (%):** NR  **Anaemia (un-specified) (%):** NR  **Weight loss (%):** NR  **Palpable mass (%):** NR  **Constipation (%):** NR  **Anaemia (iron deficiency) (%):** NR  **NICE criteria for urgent referral (%):** NR  **SIGN criteria for urgent referral (%):** NR  **Clinical suspicion of CRC (%):** NR | Sentinal Diagnostics |
| **McDonald 2013^31^**  **Related publications:**  None  **Country:** Scotland  **Funding:** Chief Scientist Office; analyser, training and consumables supplied by Mast Diagnostics Division  **Recruitment:** Feb-10 -to Mar-12  **Study design:** Diagnostic Cohort | **Inclusion criteria:** Patients (>16 years old) who had been referred from primary care for endoscopic examination of the lower gastrointestinal tract. Resons for referral included: rectal bleeding, change in bowel habit, iron deficiency  anaemia, abdominal pain, bloating, polyp/colorectal cancer surveillance, family history and assessment of IBD.  **Exclusion criteria:** Referred for investigation of a positive test result from the Scottish Bowel Screening Programme  **Study Setting:** outpatient clinic  **Point in care pathway at which the test is given:** Testing was requested after referral for endoscopy. | **Total Number tested:** 280 (Completed FIT and endoscopy)  **Age (range):** (16 - 89) years, for all invited participants  **Male (%):** 40.4  **Altered bowel habit (%):** NR  **Rectal bleeding (%):** NR  **Diarrhoea (%):** NR  **Abdominal pain (%):** NR  **Anaemia (un-specified) (%):** NR  **Weight loss (%):** NR  **Palpable mass (%):** NR  **Constipation (%):** NR  **Anaemia (iron deficiency) (%):** NR  **NICE criteria for urgent referral (%):** NR  **SIGN criteria for urgent referral (%):** NR  **Clinical suspicion of CRC (%):** NR | Eiken Chemical Co., Tokyo, Japan |
| **Mowat 2015^27^**  **Related publications:** Steele 2014  **Country:** Scotland  **Funding:**  Detect cancer early, Scottish Government  **Recruitment:**  Oct-13 to Mar-14  **Study design:**  Diagnostic Cohort | **Inclusion criteria:** All adult patients referred for investigation of bowel symptoms for any of the following reasons: rectal bleeding, anaemia, diarrhoea, altered bowel habit, abdominal pain and weight loss.  **Exclusion criteria:** NR  **Study Setting:** Primary care  **Point in care pathway at which the test is given:** Testing was ordered by GPs at the point of referring patients to secondary care  **Comments:**  Data were also presented for the diagnostic performance of individual signs and symptoms, and for the most common findings at colonoscopy | **Total Number tested:** 755 (Completed FIT and endoscopy)  **Median age (range):** 64 (16 - 90) years  **Male (%):** NR  **Altered bowel habit (%):** 42.8  **Rectal bleeding (%):** 34.2  **Diarrhoea (%):** 16.8  **Abdominal pain (%):** 11  **Anaemia (un-specified) (%):** 6  **Weight loss (%):** 0.9  **Palpable mass (%):** 0.3  **Constipation (%):** NR  **Anaemia (iron deficiency) (%):** NR  **NICE criteria for urgent referral (%):** NR  **SIGN criteria for urgent referral (%):** NR  **Clinical suspicion of CRC (%):** NR | Eiken Chemical Co., Tokyo, Japan |
| **Rodríguez-Alonso 2015^28^**  **Related publications:**  None  **Country:** Spain  **Funding:**  Societat Catalana de Digestologia, Catalonia, Spain. Instituto de SaludCarlos III, FIS grants.  **Recruitment:**  Sep-11 to Oct-12  **Study design:**  Diagnostic Cohort | **Inclusion criteria:** Adult patients (>18 years) referred for diagnostic colonoscopy; referrals were from general practitiopners, community gastroenterologists and hospital environments  **Exclusion criteria:** Adenoma surveillance; CRC surveillance; hospitalised patients; previous colectomy; IBD; polyp syndromes  **Study Setting:** Mixed  **Point in care pathway at which the test is given:** Testing was requested by the GP or at the time of the initial secondary care consultation  **Comments:** Data were also presented for the diagnostic performance of individual signs and symptoms and for NICE and SIGN referral criteria, and for findings at colonoscopy. | **Total Number tested:** 1003 (Completed FIT and endoscopy)  **Median age (range):** NR  **Male (%):** 46.8  **Altered bowel habit (%):** NR  **Rectal bleeding (%):** 34.2  **Diarrhoea (%):** 23.5  **Abdominal pain (%):** 35.4  **Anaemia (un-specified) (%):** NR  **Weight loss (%):** 19  **Palpable mass (%):** NR  **Constipation (%):** 12.1  **Anaemia (iron deficiency) (%):** 18.8  **NICE criteria for urgent referral (%):** 29.5  **SIGN criteria for urgent referral (%):** 31.2  **Clinical suspicion of CRC (%):** NR | Eiken Chemical Co., Tokyo, Japan |
| **Terhaar sive Droste 2011^34^**  **Related publications:** Oort 2011^39^; van Turenhout 2014^35^; van Turenhout 2012^38^; van Turenhout 2011^44^; Oort 2010^40^; van Turenhout 2012^36^; Larbi 2012^43^; van Turenhout 2010^45^; van Turenhout 2010^46^  **Country:** The Netherlands  **Funding:** Nycomed BV, Hoofddorp to 'the Amsterdam Gutclub.' The OC-Sensor MICRO desktop analyser was provided by Eiken Chemical Co., Tokyo, Japan  **Recruitment:** May-06 to May-10  **Study design:** Diagnostic Cohort | **Inclusion criteria:** Adult patients referred for colonoscopy  **Exclusion criteria:** No informed consent; >hemi-colectomy; IBD; age <18 years  **Study Setting:** outpatient clinic  **Point in care pathway at which the test is given:** Patients scheduled for colonoscopy were requested to perform a FIT before bowel preparation for colonoscopy  **Comments:** Data for the symptomatic subgroup were provided, AiC, by the authors (personal communication: e-mail from Sietze van Turenhout to Marie Westwood 12/06/2016) | **Total Number tested:** 2058  **Mean age (range):** 59.6 (18 - 91)  **Male (%):** 43.5  **Altered bowel habit (%):** 26.6  **Rectal bleeding (%):** 27.2  **Diarrhoea (%):** 6.2  **Abdominal pain (%):** 17.2  **Anaemia (un-specified) (%):** 11.1  **Weight loss (%):** 4.3  **Palpable mass (%):** NR  **Constipation (%):** 4.5  **Anaemia (iron deficiency) (%):** NR  **NICE criteria for urgent referral (%):** NR  **SIGN criteria for urgent referral (%):** NR  **Clinical suspicion of CRC (%):** 2.8  **Comments:** Some patients had multiple symptoms; patients were asigned to a symptom group based on the most severe symptom experienced, ie. In order of rectal bleeding, anaemia, clinical suspicion of CRC, change in bowel habit |  |
| **Thomas 2016^51^**  **Related publications:**  None  **Country:** England  **Funding:**  Reagents and FIT devices provided by industry  **Recruitment:**  Mar-15 to Mar-16  **Study design:**  Diagnostic Cohort | **Inclusion criteria:** Adult symptomatic patients, recruited after their first two week wait clinic at University Hospitals Coventry and Warwickshire NHS Trust, UK.  **Exclusion criteria:** NR  **Study Setting:** outpatient clinic  **Point in care pathway at which the test is given:** Testing was requested in secondary care. Patients were recruited after their first two week wait clinic.  **Comments:**  Abstract only | **Total Number tested:** 450  **Median age (range):**  67 (29 - 93) years  **Male (%):** NR  **Altered bowel habit (%):** NR  **Rectal bleeding (%):** NR  **Diarrhoea (%):** NR  **Abdominal pain (%):** NR  **Anaemia (un-specified) (%):** NR  **Weight loss (%):** NR  **Palpable mass (%):** NR  **Constipation (%):** NR  **Anaemia (iron deficiency) (%):** NR  **NICE criteria for urgent referral (%):** NR  **SIGN criteria for urgent referral (%):** NR  **Clinical suspicion of CRC (%):** NR | Kyowa Medex, Tokyo, Japan |

## Index test and reference standard details

| **Study Details** | **Index test details** | | | | **Reference standard details** | |
| --- | --- | --- | --- | --- | --- | --- |
|  | **Test**  **Analyser** | **Cut-offs (µg Hb/g faeces)** | **Target conditions** | **Sample collection, storage and processing** | **Reference standard details and information recorder** | **Definition of the target condition** |
| Auge 2016^33^ | HM-JACKarc  HM-JACKarc automated immunoturbidimetric analyser | >0  ≥10  ≥20  ≥30  ≥40 | Advanced neoplasia | Patients were asked to collect two consecutive bowel motions, using sample collection devices provided (Kyowa-Medex Co., Ltd., Tokyo, Japan)  Participants were asked to store samples at 4°C and return them to the laboratory with 5 days. Samples were stored at 4°C and allowed to return to room temperature befopre analysis within 24 hours  All analyses were carried out by one laboratory technician. The laboratory has a certified total quality management system and the analyser was callibrated every two weeks. | Colonoscopy.  Colonoscopy was carried out up to the caecum or obstructing carcinoma, if present. The histology of all detected lesions was evaluated by expert pathologists.  All lesions were categorised and, if polyps were detected, the polyp site was recorded and polypectomy performed wherever possible. | CRC or HRA (defined as lesions ≥1 cm or with a villous component or high grade dysplasia, or ≥3 non-advanced adenomas |
| Cubiella 2014^30^ | OC-Sensor  Automated OC-Sensor analyser | ≥20 | Colorectal cancer  Advanced neoplasia  CRC location  CRC stage | No diet or medication restrictions. Participants were instructed to collect the sample from a single faecal where no blood was visible.  No further details were reported. | Colonoscopy.  Colonoscopy was carried out by experienced endoscopists who performed >200 examinations per year. A biopsy was taken when appropriate. Colonoscopy was considered complete if caeccal intubation was achieved, unless a complete neoplastic stenosis was found.  Information recorded included: the location (rectum, colon, or proximal to the splenic flexure) and AJCC stage of any CRC; HRA (adenomas measuring ≥10 mm, with villous architecture and/or high-grade dysplasia). | Any CRC  Advanced neoplasia (CRC or HRA)  CRC (located in the rectum); CRC (located in the distal colon); CRC (located in the proximal colon)  CRC (AJCC stage 0); CRC (AJCC stage I); CRC (AJCC stage II); CRC (AJCC stage III); CRC (AJCC stage IV). |
| Godber 2016^32^ | HM-JACKarc  HM-JACKarc automated immunoturbidimetric analyser | ≥10  ≥15  ≥20  ≥25  ≥30  ≥35  ≥40 | Colorectal cancer  Advanced neoplasia  All neoplasia  Significant bowel disease | A single sample collection device (Kyowa-Medex Co., Ltd, Tokyo, Japan) was used by the patient, dated and posted back to Department of Biochemistry, Monklands Hospital, Airdrie.  Stored at 4 °C upon arrival until analysis. The mean elapsed time between sampling and receipt was 2.0 days which is within the manufacturers documented stability (stable for 120 days at 4 °C and 14 days at 25 °C). All analyses were carried out within one week of receipt in the laboratory.  All analyses were carried out by a single registered clinical scientist. The laboratory has a certified total quality management system and the analyser was callibrated once per week. | Colonoscopy.  Colonoscopy was performed by the endoscopy service at Wishaw General Hospital, which participates in the accreditation scheme of the Joint Accreditation Group on GI Endoscopy.  Data were collected on the quality of the investigation (quality of preparation, completeness) and on the results including number, size, and localisation of colorectal cancers (CRC), high and intermediate risk adenoma (higher risk adenoma: HRA), inflammatory bowel disease (IBD), colitis, low risk adenoma (LRA) and other colorectal findings and whether biopsy was performed. Pathological data were collected on all excised and biopsy specimens including polyp type, presence or absence of malignancy, stage of any cancer. | Any CRC  Advanced neoplasia (CRC or HRA); HRA was defined as three or more adenomas, or any adenoma with a diameter ≥10 mm.  All neoplasia (cancer, or HRA, or low risk adenoma)  Significant bowel disease (cancer, or HRA, or low risk adenoma, or IBD) |
| Krivec 2011^29^ | FOB Gold system  Modular Roche biochemical analyser | ≥9 | Significant bowel disease | No details reported | Colonoscopy.  No details reported | Malignant disease, polyps, or observed bleeding during endoscopy |
| McDonald 2012^31^ | OC-Sensor  OC-Sensor Diana automated immunoturbidimetric analysers. | ≥10 | Colorectal cancer  Advanced neoplasia  All neoplasia  Significant bowel disease | EIKEN specimen collection device. Patients were instructed to collect and date samples from a single faeces, and to post the sample back to the Scottish Bowel Screening Centre Laboratory immediately.  No details about sample storage were reported.  All analyses were carried out in the Scottish Bowel Screening Centre Laboratory, by staff whose main role was to perform faecal analyses. The laboratory has a certified total quality management system based on the UK Clinical Pathology Accreditation standard and analysers were callibrated once per month. | Colonoscopy and flexible sigmoidoscopy.  Colonoscopy was performed in the Endoscopy Unit.  Data were collected on the quality of the investigation (quality of preparation, completeness) and on the results including number, size and localization of colorectal cancers, HRA and low-risk adenoma, and whether biopsy was performed. Full pathological data were collected on all excised and biopsy specimens including polyp type, presence or absence of malignancy, stage of any cancer and the characteristics of all adenomas. | Any CRC  Advanced neoplasia (colorectal cancer or HRA); HRA was defined as three or more adenomas, or any adenoma with a diameter ≥10 mm.  All neoplasia (CRC, HRA, or low risk adenoma)  Significant bowel disease (cancer, or HRA, or low risk adenoma, or IBD) |
| Mowat 2015^27^ | OC-Sensor  Io Analyser | ≥10  ≥0 | Colorectal cancer  Advanced neoplasia  HRA  Significant bowel disease  Inflammatory bowel disease | EIKEN specimen collection device. Patients were instructed to collect samples from a single faeces and to return immediately to the GP surgery  The samples were returned at room temperature via the GP surgery and then stored at 4°C or -20°C prior to analysis.  The laboratory has a total quality management system in place and is accredited to ISO 15189 based standards. | Colonoscopy.  Colonoscopy was conducted in accredited endoscopy units and reported by the endoscopist. Diagnoses were confirmed following assessment by a gastrointestinal pathologist. | Any CRC  Advanced neoplasia (CRC or HRA); HRA was defined as ≥3 adenomas or any adenoma ≥10 mm.  Significant bowel disease (CRC, HRA or IBD) |
| Rodríguez-Alonso 2015^28^ | OC-Sensor  MICRO desktop analyser | ≥20  ≥15  ≥10  ≥0 | Colorectal cancer  Advanced neoplasia | EIKEN specimen collection device.  Patients were instructed to store the sample in the refridgerator at <4°C and submit it within 7 days. No detail of transportation or laboratory storage conditions were reported.  No further details were reported. | Colonoscopy.  Colonoscopy was conducted by experienced endoscopists with the patient under conscious sedation. The colonoscopy was considered complete if caecal intubation was achieved, as demonstrated by visualisation of the ileocecal valve or the appendiceal orifice. The  number, size and histology of polyps, and presence or absence of CRC were recorded. | Any CRC  Advanced neoplasia (CRC or HRA) |
| Terhaar sive Droste 2011^34^ | OC-Sensor  MICRO desktop analyser | ≥10  ≥15  ≥20  ≥30  ≥40 | Colorectal cancer  HRA | Patients were asked to collect a faecal sample the day before colonoscopy and before bowel preparation had started. Illustrated guidance was provided on how to collect a sample without exposure to water or urine. No dietary or medication restrictions were required.  Samples were submitted on the day of colonoscopy, stored at -5°C, and analysed within one week of collection.  Samples were analysed by two experienced technicians. | Colonoscopy.  Colonoscopy was conducted in accredited endoscopy units and reported by the endoscopist. Diagnoses were confirmed following assessment by a gastrointestinal pathologist. A complete colonoscopy was defined as intubation of the caecum with identification if the ileocecal valve or appendiceal orifice, or intubation up to an obstructing neoplasm | Any CRC  HRA was defined as >9 mm, high grade dysplasia and/or villous adenoma. |
| Thomas 2016^51^ | HM-JACKarc  HM-JACKarc automated immunoturbidimetric analyser | ≥7 | Colorectal cancer  Significant bowel disease | No details reported. | CT/ Colonoscopy.  No further details reported. | Significant bowel disease (CRC, HRA or IBD) |
| CRC = colorectal cancer; Hb = haemoglobin; HRA = high risk adenoma; AJCC = American Joint Committee on Cancer; IBD = inflammatory bowel disease; CT = computed tomography; GI = gastrointestinal; GP = general practitioner | | | | | | |
