# Supplementary material for: Faecal immunochemical tests (FIT) can help to rule out colorectal cancer in patients presenting in primary care with lower abdominal symptoms: a systematic review conducted to inform new NICE DG30 diagnostic guidance
Source: BMC Med. 2017 Oct 24;15:189. doi: 10.1186/s12916-017-0944-z (PMC5654140; doi:10.1186/s12916-017-0944-z)
Supplement: Supplementary file 5 — PROBAST results for studies reporting the development and validation of risk scores that included FIT (DOCX 16 kb) [file 12916_2017_944_MOESM5_ESM.docx]

**Table S4: PROBAST results for studies reporting the development and validation of risk scores that included FIT**

| **Study** | **RISK OF BIAS** | | | | | | | | | | | **APPLICABILITY CONCERNS** | | | | | | |
| --- | --- | --- | --- | --- | --- | --- | --- | --- | --- | --- | --- | --- | --- | --- | --- | --- | --- | --- |
|  | **PARTICIPANT SELECTION** | | **PREDICTORS** | | **OUTCOME** | | **SAMPLE SIZE AND PARTICIPANT FLOW** | | **ANALYSIS** | | **OVERALL JUDGEMENT** | **PARTICIPANT SELECTION** | | **PREDICTORS** | | **OUTCOME** | | **OVERALL JUDGEMENT** |
|  | Development | Validation | Development | Validation | Development | Validation | Development | Validation | Development | Validation |  | Development | Validation | Development | Validation | Development | Validation |  |
| Cubiella 2016^30^ | Low | Low | High | High | Low | High | Unclear | Unclear | Unclear | Unclear | High | High | High | High | High | Low | Low | High |
| Rodríguez-Alonso 2015^28^ | Low | Low | Unclear | Unclear | Low | Low | Unclear | Unclear | High | High | High | High | High | High | High | Low | Low | High |
