# Supplementary material for: Faecal immunochemical tests (FIT) can help to rule out colorectal cancer in patients presenting in primary care with lower abdominal symptoms: a systematic review conducted to inform new NICE DG30 diagnostic guidance
Source: BMC Med. 2017 Oct 24;15:189. doi: 10.1186/s12916-017-0944-z (PMC5654140; doi:10.1186/s12916-017-0944-z)
Supplement: Supplementary file 6 — Sub-group data for HM-JACKarc. (DOCX 19 kb) [file 12916_2017_944_MOESM6_ESM.docx]

**TABLE S5: SUB-GROUP DATA FOR HM-JACKarc**

**Effects of participant sex on the accuracy of HM-JACKarc for the detection of advanced neoplasia (CRC or HRA) using a single faecal sample**

| **Study** | **Subgroup** | **Faecal haemoglobin cut-off**  **(µg Hb/g faeces)** | **True Positive** | **False Negative** | **False Positive** | **True Negative** | **Total** | **Sensitivity % (95% CI)** | **Specificity % (95% CI)** |
| --- | --- | --- | --- | --- | --- | --- | --- | --- | --- |
| Auge 2016^33^ | Men | ≥10 | 8 | 9 | 10 | 65 | 92 | 47.1 (26.2, 69.0) | 86.7 (77.2, 92.6) |
|  | Women | ≥10 | 2 | 10 | 13 | 91 | 116 | 16.7 (4.7, 44.8) | 87.6 (79.8, 92.6) |
|  | Men | ≥20 | 7 | 10 | 6 | 69 | 92 | 41.2 (21.6, 64.0) | 92.0 (83.6, 96.3) |
|  | Women | ≥20 | 2 | 10 | 7 | 97 | 116 | 16.7 (4.7, 44.8) | 93.3 (86.8, 96.7) |
|  | Men | ≥30 | 7 | 10 | 6 | 69 | 92 | 41.2 (21.6, 64.0) | 92.0 (83.6, 96.3) |
|  | Women | ≥30 | 2 | 10 | 6 | 98 | 116 | 16.7 (4.7, 44.8) | 94.3 (88.0, 97.3) |
|  | Men | ≥40 | 7 | 10 | 6 | 69 | 92 | 41.2 (21.6, 64) | 92.0 (83.6, 96.3) |
|  | Women | ≥40 | 1 | 11 | 5 | 99 | 116 | 8.3 (1.5, 35.4) | 95.2 (89.3, 97.9) |

Effects of multiple sampling on the accuracy of HM-JACKarc for the detection of advanced neoplasia (CRC or HRA)

| **Study** | **Sampling strategy** | **Faecal haemoglobin cut-off**  **(µg Hb/g faeces)** | **True Positive** | **False Negative** | **False Positive** | **True Negative** | **Total** | **Sensitivity % (95% CI)** | **Specificity % (95% CI)** |
| --- | --- | --- | --- | --- | --- | --- | --- | --- | --- |
| Auge 2016^33^ | First of two consecutive samples | ≥10 | 10 | 19 | 23 | 156 | 208 | 34.5 (19.9, 52.7) | 87.2 (81.6, 91.3) |
|  | Highest of two consecutive samples | ≥10 | 12 | 17 | 37 | 142 | 208 | 41.4 (25.5, 59.3) | 79.4 (73, 84.7) |
|  | First of two consecutive samples | ≥20 | 9 | 20 | 13 | 166 | 208 | 31.0 (17.3, 49.2) | 92.8 (88.0, 95.7) |
|  | Highest of two consecutive samples | ≥20 | 10 | 19 | 26 | 153 | 208 | 34.5 (19.9, 52.7) | 85.6 (83.5, 92.9) |
|  | First of two consecutive samples | ≥30 | 9 | 20 | 12 | 167 | 208 | 31.0 (17.3, 49.2) | 93.3 (88.7, 96.1) |
|  | Highest of two consecutive samples | ≥30 | 10 | 19 | 25 | 154 | 208 | 34.5 (19.9, 52.7) | 86.1 (83.6, 92.9) |
|  | First of two consecutive samples | ≥40 | 8 | 21 | 11 | 168 | 208 | 27.6 (14.7, 45.7) | 93.9 (89.4, 96.6) |
|  | Highest of two consecutive samples | ≥40 | 10 | 19 | 21 | 158 | 208 | 34.5 (19.9, 52.7) | 88.3 (82.8, 92.2) |
